# Novel Antibody Drug Conjugates Targeting Tumor-Associated Receptor Tyrosine Kinase ROR2 by Functional Screening of Fully Human Antibody Libraries Using Transpo-mAb Display on Progenitor B Cells

**DOI:** 10.3389/fimmu.2018.02490

**Published:** 2018-11-02

**Authors:** Ina Hellmann, Lorenz Waldmeier, Marie-Christine Bannwarth-Escher, Kseniya Maslova, Fabian I. Wolter, Ulf Grawunder, Roger R. Beerli

**Affiliations:** NBE-Therapeutics Ltd., Basel, Switzerland

**Keywords:** antibody drug conjugate (ADC), transposition, mammalian IgG cell display, antibody discovery, functional screen, ROR2, human immune library, human IgG transgenic mice

## Abstract

Receptor tyrosine kinase-like orphan receptor 2 (ROR2) has been identified as a highly relevant tumor-associated antigen in a variety of cancer indications of high unmet medical need, including renal cell carcinoma and osteosarcoma, making it an attractive target for targeted cancer therapy. Here, we describe the *de novo* discovery of fully human ROR2-specific antibodies and potent antibody drug conjugates (ADCs) derived thereof by combining antibody discovery from immune libraries of human immunoglobulin transgenic animals using the Transpo-mAb mammalian cell-based IgG display platform with functional screening for internalizing antibodies using a secondary ADC assay. The discovery strategy entailed immunization of transgenic mice with the cancer antigen ROR2, harboring transgenic IgH and IgL chain gene loci with limited number of fully human V, D, and J gene segments. This was followed by recovering antibody repertoires from the immunized animals, expressing and screening them as full-length human IgG libraries by transposon-mediated display in progenitor B lymphocytes (“Transpo-mAb Display”) for ROR2 binding. Individual cellular “Transpo-mAb” clones isolated by single cell sorting and capable of expressing membrane-bound as well as secreted human IgG were directly screened during antibody discovery, not only for high affinity binding to human ROR2, but also functionally as ADCs using a cytotoxicity assay with a secondary anti-human IgG-toxin-conjugate. Using this strategy, we identified and validated 12 fully human, monoclonal anti-human ROR2 antibodies with nanomolar affinities that are highly potent as ADCs and could be promising candidates for the therapy of human cancer. The screening for functional and internalizing antibodies during the early phase of antibody discovery demonstrates the utility of the mammalian cell-based Transpo-mAb Display platform to select for functional binders and as a powerful tool to improve the efficiency for the development of therapeutically relevant ADCs.

## Introduction

Cancer is still a leading cause of death worldwide. While the landscape of cancer treatment has positively evolved recently with the advent of targeted, including antibody- and cell-based therapies, broadly effective and curative treatment options still remain limited. Classical chemotherapy with cytotoxic or cytostatic small molecules remains the standard of care in many anti-cancer treatments, although dose-limiting toxicities as well as limited selectivity against cancer cells result in only partial clinical efficacy (1). To increase the anti-tumor efficacy and to lower the toxicity on normal tissues, a targeted delivery of the cytotoxic agent to the tumor has long been desired. With the development of monoclonal antibodies (mAbs) (2), it became a possibility to use their specific binding to a tumor-associated antigen (TAA) to specifically target cancer cells. Naked antibodies either need to have an intrinsic capability to interfere with the growth of cancer cells, or they need to recruit other immune-system components to inhibit tumor growth and expansion. These activities of antibodies are often insufficient to effect complete eradication of targeted tumor cells. Therefore, selectively delivering a highly cytotoxic substance to the tumor by generating antibody drug conjugates (ADCs) has been considered an attractive concept for decades. ADCs consist of an antibody conjugated via a covalent linker to a potent cytotoxic payload, thereby combining the high selectivity of the antibody moiety to a TAA and the otherwise intolerably high cytotoxic potential of the payload (3). However, in order to generate not only potent, but also safe ADCs, many aspects of the molecule need to be optimal. This includes not only the tumor-selective binding of the antibody moiety to the targeted cancer cells, but also functional properties of the antibody moiety, such as its ability to internalize the ADC into the targeted cancer cell following binding of the ADC to the TAA. While the concept of antibody-mediated delivery of toxic payloads to cancer cells is known in the field for decades, due to the structural complexity of this class of molecules and the high functional requirements to achieve a favorable therapeutic index, only four ADCs have been approved to date by the regulatory authorities for treating cancer patients, while >60 ADCs are currently evaluated in clinical trials (4). In the field of targeted cancer therapies, ADCs will have an enormous potential for cancer treatment in the future, if engineered diligently with regard to linker stability and product homogeneity as well as optimal tumor selectivity and functionality.

It was the objective of this study to discover antibodies specifically targeting the TAA ROR2 with high internalization rates, eventually leading to potent cancer cell killing with anti-ROR2-antibody based ADCs. ROR2, along with ROR1, belongs to the receptor tyrosine kinase-like orphan receptor (ROR) family, both type I transmembrane proteins with a high degree of structural homology (5). The structure of both ROR1 and ROR2 includes an extracellular portion with three distinct domains, an N-terminal Ig-like domain, a Frizzled domain (or cysteine-rich repeat domain, CRD) and a membrane-proximal Kringle domain, followed by a transmembrane domain connecting to intracellular domains with a structural and amino acid homology to receptor tyrosine kinases (6, 7). Here, we focused on human ROR2 and the identification of novel mAbs and ADCs against this promising cancer cell target. Like ROR1, ROR2 regulates cellular processes including cell proliferation, polarity, differentiation, migration, metabolism and survival (8–10). While ROR2 is expressed in a wide variety of tissues during early embryogenesis (11), it is mainly absent in adult normal tissues (7). In contrast, ROR2 is overexpressed in several human cancers, including renal cell carcinoma (11, 12), osteosarcoma (10), melanoma (13), stromal tumors (14), as well as breast (15, 16), colorectal (17), oral (18), and pancreatic cancer (19), and has been associated with a more aggressive disease state and poorer patient prognosis within these indications. Recently, it has been shown that ROR2 interacts with ligands Wnt5a and Wnt3a to activate a combination of noncanonical and canonical Wnt signaling pathways, respectively (9, 20–22). Upregulation of ROR2 is associated with mediating pro-tumorigenic activity (polarized cell migration, invasion, and tumor growth) via the noncanonical Wnt signaling pathway (7, 15, 23). In addition to its function as an oncogene, and in contrast to ROR1, ROR2 can also act as a suppressor of carcinogenesis in tumors driven by canonical Wnt signaling, where ROR2 expression is lost, as observed in colon cancer and hepatocellular carcinoma (6). Notably, the expression of ROR2 is largely mutually exclusive with its sister molecule ROR1 and only rare cases of ROR1 and ROR2 co-expression on tumors have been reported (5, 24).

To develop potent ADCs, antibodies with selectivity for the TAA and triggering high internalization rates are generally needed (4). This is particularly relevant for TAAs for which the level of expression on cancer cells is not extraordinarily high, like in the case of ROR2 (5, 6). The mode of action of ADCs requires specific binding to the TAA, upon which the ADC-TAA complex needs to be internalized by receptor-mediated endocytosis and directed toward lysosomal degradation, where the toxic payload is released intracellularly (25). Antibodies may vary in the rate and magnitude of internalization they are inducing, even if the same epitope on the cancer target antigen is recognized (26, 27). Hence, it is desirable to identify antibodies that are potent ADCs during early discovery. While antibody discovery technologies in eukaryotic cells are manifold, spanning from yeast (28, 29) to mammalian cell-based antibody expression platforms (30–33), these platforms typically do not allow a rapid and efficient screening for desired functional properties, e.g., potency as an ADC, without the need for cloning and re-formatting of the antibody into a soluble form. In addition, many antibody discovery technologies yield non-human antibodies that require tedious humanization of the antibody in order to lower immunogenicity which could affect clinical safety and efficacy of the therapeutic (34, 35). Hence, a straightforward antibody screening platform to quickly identify functionally relevant fully human antibodies is highly desired.

In the present study, we describe the *de novo* discovery of fully human antibodies and potent ADCs derived thereof targeting the highly tumor selectively expressed human ROR2, using the Transpo-mAb Display (36) of immune antibody libraries isolated from immunized transgenic mice harboring transgenic IgH and IgL gene loci with human V, D, and J gene segments. The straightforward functional screening of clonal supernatants from fully human immune cellular libraries allows for identification of mAbs suitable for the development of potent ADCs already during the early stages of antibody screening.

## Materials and methods

### Cell lines

The origin and culturing conditions of murine progenitor B cell clone L11 derived from the Abelson-murine leukemia virus transformed progenitor B cell line 63-12 of a RAG2-deficient mouse and the murine breast cancer cell line EMT6 have been previously described (36). Human multiple myeloma cell line L363 (DSMZ) and human breast cancer cell line T47D (ATCC) were cultured in RPMI or DMEM, respectively, supplemented with 10% fetal calf serum (FCS), 2 mM L-glutamine, 100 IU penicillin, 0.1 mg/ml streptomycin and 0.25 μg/ml fungizone (all from Amimed) at 37°C in a humidified incubator at 5% CO_2_ atmosphere.

### Mouse strains

H2L2 mice were obtained from Harbor Biomed; H2L2 mice are a cross of the following mouse strains: F129, fvb/n and C57BL6, and upon immunization produce antibodies with a human variable domain and a rat constant domain, disclosed in patent application WO 2010/070263A1. Experimental procedures involving mice were performed at certified animal facilities at ETH-D-BSSE, Basel. All procedures involving animals were compliant with the guidelines and protocols for animal care and handling approved by the Basel-Stadt cantonal veterinary office.

### Immunization

Homozygous transgenic mice harboring transgenic IgH and IgL gene loci with a limited number of fully human V, D, and J gene segments back-crossed on IgH and IgκL chain double-knock-out background and designated H2L2 mice were obtained from Harbor BioMed in Cambridge, Massachusetts. While the V, D, and J gene segments in the transgenic IgH chain gene loci were fully human sequences, the coding region of the constant region exons were of rat origin, such that the mice primarily generate chimeric antibodies encoded by fully human V_H_ and V_L_ regions and rat constant regions. C57BL/6 wild-type mice were obtained from Janvier Laboratories (Saint-Berthevin Cedex, France). Mice were immunized with a soluble human ROR2 extracellular domain (ECD) fused to a Twin-Strep-tag (hROR2-ECD-Twin-Strep) using the following schedule: On day 0, mice were immunized intraperitoneally using 50 μg hROR2-ECD-Twin-Strep diluted in PBS and 20 μg monophosphoryl lipid A (MPLA) (Invivogen, tlrl-mpls) mixed 1:1 with Addavax adjuvant (Invivogen, vac-adx-10), creating an oil-in-water emulsion in 100 μl. On day 21, mice were boosted intraperitoneally using 20 μg hROR2-ECD-Twin-Strep diluted in PBS and 20 μg MPLA mixed 1:1 with Addavax adjuvant in 100 μl. On day 42, the mice were boosted intravenously using 10 μg hROR2-ECD-Twin-Strep without any adjuvants added in 100 μl. Blood sampling was performed from the tail vein of mice 7 days prior to the first immunization, and 7 days following each immunization (days 7, 28), or by heart puncture on day 49, when the mice were sacrificed. Blood was allowed to clot for 15–60 min at room temperature, then spun down at 5,000 rpm for 15 min at 4°C. Serum was carefully transferred into a new tube and stored at −20°C until further use.

On day 49 following initial antigen injection, the mice were euthanized, spleens were collected, transferred into 1x PBS and stored on ice until processing. Each spleen was homogenized in a gentleMACS C-tube (Miltenyi Biotec) containing 2.4 ml of RPMI-10%FCS using the gentleMACS Octo Dissociator (Miltenyi Biotec). Cells were then filtered through a cell strainer to get a single cell suspension, frozen down in 90% FCS/10%DMSO, and stored in the vapor phase of liquid nitrogen until further use.

### Library construction

For the isolation of antigen-specific B cells, splenocytes were thawed quickly at 37°C and washed twice in cold MACS buffer (0.5% BSA in PBS, 2 mM EDTA). Cell viability was assessed using 0.4% Trypan Blue (Amresco). Between 1.9 × 10^6^ and 3.7 × 10^6^ total viable cells were incubated with 20 μg/ml mouse IgG (ChromPure Mouse IgG, 015-000-003, Jackson Immunoresearch) for 15 min at 4°C. Isolation of antigen-specific B cells was performed in two consecutive steps: depletion of non-B cells, followed by positive selection for antigen-specific B cells. Magnetic labeling of non-B cells was performed using the Pan B Cell Isolation Kit Mouse (Miltenyi Biotec), according to the manufacturer's protocol. For the positive selection, 50 μl Strep-Tactin Magnetic Nanobeads (IBA Lifesciences) were coated with 12 μg hROR2-ECD-Twin-Strep over night at 4°C, followed by washing with MACS buffer in a magnetic field to remove unbound antigen and elution. Next, isolated B cells were incubated with hROR2-ECD-Twin-Strep-coated magnetic beads for 45 min, washed with MACS buffer and subjected to magnetic separation using LS columns (Miltenyi Biotec). The flow-through was discarded and elution was performed after removal from the magnetic field, yielding an enrichment of antigen-positive B cells. Cells were centrifuged and directly subjected to RNA extraction.

RNA was isolated using TRI-Reagent (Sigma-Aldrich) and reverse transcribed using Protoscript II Reverse Transcriptase (New England Biolabs) using random nonamers, following the manufacturer's instructions. Variable domains were amplified by semi-nested PCR using Q5 DNA polymerase (New England Biolabs). In general, in the first step of this PCR, a set of forward primers binding to the 5′-end of the framework (FR) 1 of human variable domains, thereby adding the 3′-portion of a universal leader peptide to the 5′-end of the amplicon, was paired with a reverse primer specific to the rat constant domain expressed in H2L2 mice. In the second PCR-step, a forward primer completing the N-terminal leader peptide and adding a restriction site for cloning at the 5′-end of the amplicon was paired with a set of reverse primers binding to the 3′-end of the FR4, thereby adding a restriction site for cloning to the 3′-end of the amplicon. The resulting amplicons contained the nucleotide sequences of restriction sites for cloning at the 5′-end and 3′-end, as well as a complete leader sequence followed by the entire human variable V(D)J domain. The primer sequences are supplied in the Supplementary information (Figure S1).

In the first PCR step, human IgG and IgM heavy chain (HC) variable domains were amplified using human V_H_-specific forward primers (primer set: Set-huHC-FR1) paired with a reverse primer specific for the rat IgG (rat_IgG12abc_R) or IgM constant domain (rat_IgM_R). In the 2nd PCR step, forward primer Not-I-5′Leader and reverse primers specific to the human J_H_-domain (primer set: Set-huJH-R) were used to complete the PCR amplification. For amplification of Igκ light chain (LC) variable domains, a forward primer set specific to human V_κ_-domains (primer set: Set-huKC-FR1) was paired with a reverse primer binding to the rat IgκLC constant domain (rat_CK_R) in the 1st PCR step. For the 2nd PCR step, forward primer Not-I-5′-Leader was paired with a primer set specific to human J_k_-domains (primer set: Set-huJK-R) to generate the final amplicon. In H2L2 mice, the endogenous mouse lambda light chain gene loci have not been knocked-out and remain intact, and no human lambda light chains are expressed. Therefore, the lambda light chain gene loci were not amplified. Cycling conditions were as follows: 98°C/30s -> 15-25x (98°C/10 s, 56°C/30 s, 72°C/60 s) ->72°C, 2 min -> hold@ 4°C. For the first and second PCR, 25 or 15 cycles were performed, respectively.

Amplicons were then cloned into transposable vectors as previously described (36). In brief, IgG- and IgM-derived V_H_ domains were cloned into pPB-Hygro-HCγ1-gen IgHC expression vector, encoding a genomic human IgG_1_ constant domain, while kappa LC variable domains were cloned into pPB-Puro-LC, thereby complemented with a human Kappa constant domain, via NotI/NheI or NotI/BsiWI, respectively. Libraries were ligated, transformed into XL1-Blue electrocompetent cells, and at least 10 bacterial clones were analyzed by sequencing, as previously described (36). Library sizes ranged from 6 × 10^6^ to 5 × 10^7^ independent transformants.

Antibody protein sequences were annotated and assessed for their degree of identity to the closest human or mouse germline V gene sequence with using IgBLAST (https://www.ncbi.nlm.nih.gov/igblast/).

### Transposition, staining and sorting of cellular libraries

63-12 murine progenitor B cells, L11 cells, were transposed using a HC:LC:Transposase DNA weight ratio of 0.25:0.125:1, and selected using 1 μg/ml puromycin and 800 μg/ml hygromycin B, as previously described (36).

For staining and sorting of cellular libraries, 0.2–1 × 10^7^ cells were stained with hROR2-ECD-Twin-Strep at concentrations between 0.25 and 2 μg/ml, Strep-MAB Oyster Classic 645 (IBA Lifesciences, 2-1555-050) diluted 1:500 and PE-labeled anti-human IgG (Fcγ-specific; ebioscience, 12-4998-82) diluted 1:250 in cold 2% FCS in PBS for 1 h on ice. For control stainings without antigen, hROR2-ECD-Twin-Strep was omitted in the staining. Following washing with 2% FCS in PBS, cells were either analyzed by flow cytometry using a FACSCalibur Instrument (Becton-Dickinson) or filtered using a cell strainer snap cap FACS tube and single-cell sorted using a FACSAriaII instrument (Becton-Dickinson) with data analysis being performed using FlowJo analytical software (Tree Star, Ashland, OR). 288 hROR2-positive L11 clones per library were single-cell sorted into warm SF-IMDM medium supplemented with 2% FCS, 2 mM L-glutamine, 100 IU penicillin, 0.1 mg/ml streptomycin (all from Amimed), and 50 μM beta-mercaptoethanol (Amresco) at 37°C and 5% CO_2_ atmosphere.

### Antigen expression of cell lines

For determination of antigen expression on cell lines, 1 × 10^6^ cells were stained with 2 μg/ml hROR2-specific antibody XBR2-401 (37) for 30 min on ice, detected using a PE-labeled anti-human IgG (Fcγ-specific) at 1:250 dilution for 30 min on ice and analyzed by flow cytometry using a FACSCalibur instrument (Becton-Dickinson), followed by data analysis using FlowJo analytical software (Tree Star, Ashland, OR).

### ELISA

For determination of serum titers and antigen-binding, Nunc-Immuno MaxiSorp 96-well plates (Thermo Fisher Scientific) were coated with 2 μg/ml antigen diluted in coating buffer (100 mM bicarbonate/carbonate buffer). For determination of IgG titers, plates were coated with 2 μg/ml AffiniPure donkey anti-human IgG F(ab′)_2_ fragment, Fcγ-specific (109-006-008) or AffiniPure goat anti-human IgG F(ab′)_2_ fragment, Fcγ-specific (109-006-098, both Jackson Immunoresearch) diluted in coating buffer overnight at 4°C. Plates were then washed twice with 0.05% Tween-20 in PBS (PBS-T), blocked at 37°C using PBS-T supplemented with 3% bovine serum albumin (BSA) (Carl Roth) for 1 h and washed again twice with PBS-T. Serum of the H2L2 and C57BL/6 mice was pre-diluted 1:100 in PBS-T/1% BSA. L11 clonal supernatants were pre-diluted 5-fold in PBS-T/1% BSA. Supernatants from transiently transfected HEK293T cells were pre-diluted 50-fold. Purified monoclonal antibodies were used at a starting concentration between 0.5–2 μg/ml. Samples were added to plates as serial dilutions (2.5-fold for serum titer; 3-fold for L11 and HEK293T cell supernatants; 3 to 20-fold for purified monoclonal antibodies) in PBS-T/1% BSA and were incubated for 1 h at 37°C. After six washes with PBS-T, horse radish peroxidase (HRP)-conjugated F(ab′)_2_ anti-human Fcγ (109-036-008, Jackson Immunoresearch) diluted 10,000-fold in PBS-T/1% BSA buffer was added, and plates were incubated for 1 h at 37°C. For assessment of serum titers in H2L2 and C57BL/6 mice, HRP-conjugated F(ab′)_2_ anti-rat Fcγ (112-036-071) or HRP-conjugated F(ab′)_2_ anti-mouse Fcγ (115-036-071, both Jackson Immunoresearch) was diluted 5,000-fold in PBS-T/1% BSA, respectively. After washing six times with PBS-T, Sigmafast OPD Peroxidase substrate (Sigma-Aldrich) was added, and reactions were stopped by adding 2 M H_2_SO_4_. Absorption was measured at 490 nm using a Spark^TM^ 10M (Tecan Life Sciences). Half-maximal concentrations (EC50) values of standards with known concentrations and unknown samples were determined by 4-parameter curve fitting models in GraphPad Prism (GraphPad Software Inc.).

### *In vitro* potency assays

For the secondary ADC assay, 1,000 EMT6-hROR2 cells engineered to overexpress hROR2 were plated per well in 96-well plates. On the next day, clonal L11 cell supernatants were added undiluted with a final dilution on the plate of 10-fold (for single-well secondary killing assays) or using a 2-fold serial dilution using an IgG starting concentration adjusted to the IgG levels of the lowest expressing L11 clone (as determined by ELISA) for a titration curve assay, while control antibodies were 2-fold serially diluted resulting in a final concentration on the plate ranging from 1 μg/ml to 0.5 ng/ml. Following incubation at 37°C for 30 min, PNU-159682-coupled secondary ADC (HFc-CL-PNU, Moradec AH-102PN) was added with a final concentration of 0.5 nM and incubated for 3 days at 37°C.

For *in vitro* potency assays using purified ADCs, 1,000 hROR2-overexpressing T47D-hROR2 cells, or 10,000 L363 cells per well were plated in 96 well-plates. The following day ADC was added in 3.5-fold serial dilutions, with the final concentrations ranging from 20,000 to 0.25 ng/ml in duplicate, and incubated for 4 days at 37°C.

Following incubation at 37°C, cell viability was assessed using CellTiter-Glo® 2.0 Luminescence Assay (Promega G9243), according to the manufacturer's instructions, using a Tecan Infinite F200 or a Spark^TM^ 10M (both Tecan Life Sciences) with an integration time of 250 ms per well. IC_50_ values were determined by 4-point curve fitting models in GraphPad Prism (GraphPad Software Inc.).

### Expression and purification of antibodies and antigens

Human V_H_ and V_L_ sequences recovered from L11 cell clones were obtained by sequence recovery as previously described and assembled into the pCB14b or pCB14g vector with the respective constant domains (36). The resulting antibody sequences contained a sortase A recognition motif and a Twin-Strep-tag as described previously (38) for subsequent conjugation to toxin. These tag sequences were: IgH chain, (LPETG-G-WSHPQFEK(G_3_S)_3_AWSHPQFEKGS); Igκ chain, G_4_S-LPETG-G-WSHPQFEK(G_3_S)_3_AWSHPQFEKGS). In cases where more than one V_H_ and/or V_L_ were found, a deconvolution step was performed to identify the correct pairing, by expressing all possible HC/LC combinations in HEK293T cells and evaluating hROR2-ECD-Twin-Strep-binding by ELISA.

The antigens hROR2-ECD-Twin-Strep, cynomolgus monkey ROR2-ECD-Twin-Strep and mouse ROR2-ECD-Twin-Strep comprise the extracellular domain of human ROR2 (NP_004551.2, amino acids 1-403), cynomolgus monkey ROR2 (XP_005582291.1, amino acids 1-403) or mouse ROR2 (NP_038874.3, amino acids 1-402), respectively, fused at the C-Termini to a Twin-Strep-tag (GSWSHPQFEK(G_3_S)_2_G_2_SAWSHPQFEKGS) for purification. The corresponding nucleotide sequences of the respective antigens flanked with 5′-NotI and 3′-BstBI sites were produced by total gene synthesis (GenScript), cloned into the expression vector pCB14b (36) and confirmed by DNA sequencing.

Transient and semi-stable expression of antibodies in HEK293T cells using Lipofectamine® LTX with Plus™ Reagent (Thermo Fisher Scientific), followed by FPLC-based purification using Amsphere^TM^ Protein A columns (JSR Life Sciences) and Protein A HiTrap columns (GE Healthcare) on an ÄKTA pure (GE Healthcare) were performed as previously described (36).

For purification of antigens, harvested supernatants were subjected to FPLC-based affinity purification using Strep-Tactin columns (IBA Lifesciences), according to the manufacturer's protocol.

### Sortase-mediated antibody conjugation (SMAC)

LPETG-tagged antibodies were site-specifically conjugated to glycine-modified toxins Gly_3_-EDA-PNU or Gly_5_-EDA-PNU using sortase-enzyme mediated antibody conjugation (SMAC-technology^TM^), as previously described (38, 39). hROR2-specific ADCs were formulated in PBS. The drug-to-antibody-ratio (DAR) of the final ADCs ranged between 3 and 4, as determined by HPLC (38, 39).

### Surface plasmon resonance (SPR)

Affinities of anti-hROR2 antibodies to hROR2-ECD were measured by multi-cycle SPR on a Biacore T200 instrument (GE Healthcare), as described (36). Antibodies were captured using a CM5 Protein A chip (GE-Healthcare 29127556) or by Protein G immobilized on a CM5 sensor chip. hROR2-ECD-Twin-Strep was diluted in running buffer using 2-fold serial dilutions ranging from 40 nM to 2.5 nM. Capture levels ranged from 148 to 845 RU.

## Results

### Outline of functional screening strategy for direct identification of novel mAbs with optimal ADC activity

**Figure 1:**
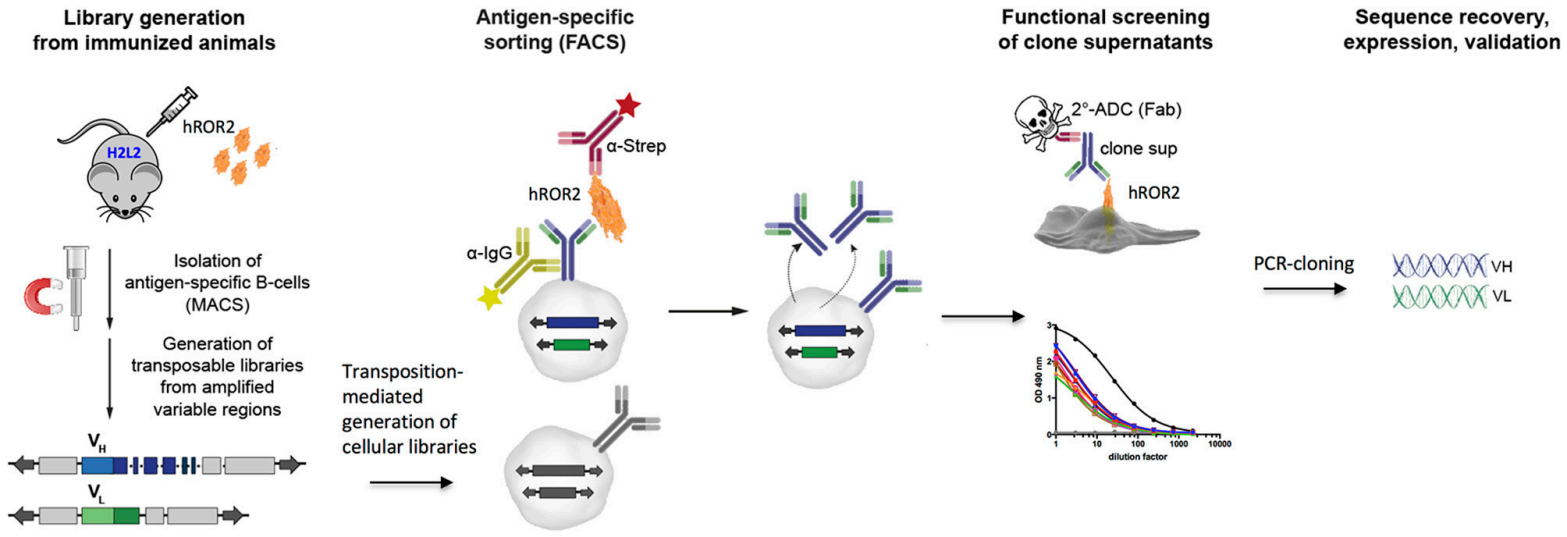
Graphic representation of the discovery strategy for novel, fully human mAbs and functional ADCs from immune libraries of Ig transgenic mice. H2L2 Ig transgenic mice (Harbor BioMed) capable of generating antibodies with completely human V_H_ and V_L_ regions were immunized with soluble human ROR2-ECD-Twin-Strep. Transpo-mAb compatible libraries were generated from MACS-enriched antigen-specific splenocytes and co-electroporated into L11 mouse pro-B cells (with a RAG2-knock-out background) employing Piggy-Bac transposase to allow for stable integration. Cell clones expressing antigen-specific antibodies were single-cell sorted by FACS. Supernatants of these single-cell clones were directly screened for antigen-binding by ELISA and their suitability as an ADC by a secondary, functional ADC-based cell killing assay. Potent ADC candidates were subjected to sequence recovery of variable domain coding regions by RT-PCR and validated following expression of the antibodies in HEK293T cells.

We applied the Transpo-mAb mammalian cell IgG Display platform (36) (in short “Transpo-mAb Display”) on immune libraries from Ig-transgenic mice with human V_H_ and V_L_ regions, to identify novel monoclonal antibodies against the extracellular domain of human ROR2 while concomitantly screening these antibodies for their suitability as ADCs without the need for prior sequence recovery, re-cloning and re-expression. An overview of the functional screening strategy is shown in Figure 1. Briefly, immunoglobulin transgenic mice expressing antibodies with fully human V_H_ and V_L_ sequences (called H2L2 mice), provided by Harbor BioMed, Cambridge, MA, were immunized with the extracellular domain of human ROR2 containing a C-terminal Twin-Strep-tag (hROR2-ECD-Twin-Strep). hROR2-specific B lymphocytes were enriched from the spleens of immunized mice using magnetic activated cell sorting (MACS), RNA was isolated from hROR2-ECD-Twin-Strep enriched B lymphocytes and libraries of coding regions for human V_H_ and kappa V_L_ variable domains were amplified by RT-PCR using specific primers. The variable region fragments were then cloned into separate transposable expression vectors for IgH and IgL chains in which the cloned V_H_ and V_L_ regions were fused with IgG_1_ HC and kappa LC constant regions, respectively, thus allowing for expression of fully human IgG_1_/κLC antibodies. The HC vector comprises a genomic version of the human HCγ 1 constant region including the two exons for the IgG membrane anchor and intracellular regions with complete intron sequences, thus allowing alternative splicing of a primary Ig-heavy chain transcript in certain B-lineage cells to allow for co-expression of high levels of both, membrane-bound and secreted antibody from the same expression vector (36). Following DNA library construction, cellular libraries were generated by stable transposition of HC and LC transposable vectors to stably display fully human IgG_1_ antibodies on the surface of immortalized murine L11 pro-B cells derived by subcloning from the A-MuLV transformed RAG-2 knock-out cell line 63-12 (36), unable to express endogenous murine Ig components. Cells expressing hROR2-specific IgG were isolated by FACS using double staining for IgG and hROR2 binding followed by single-cell sorting of hROR2-binding cellular clones using FACS. Supernatants from single-cell sorted Transpo-mAb Display cell clones containing secreted antibodies were then used to perform functional screening for their binding to hROR2 by ELISA as well as their suitability as ADCs in a secondary cell killing assay. Sequences were only recovered upon confirmation of mAbs that showed efficient cell killing in the secondary ADC cell killing assay.

### Immunization of H2l2 Ig transgenic and C57BL/6 wild-type mice with human ROR2-ECD

**Figure 2:**
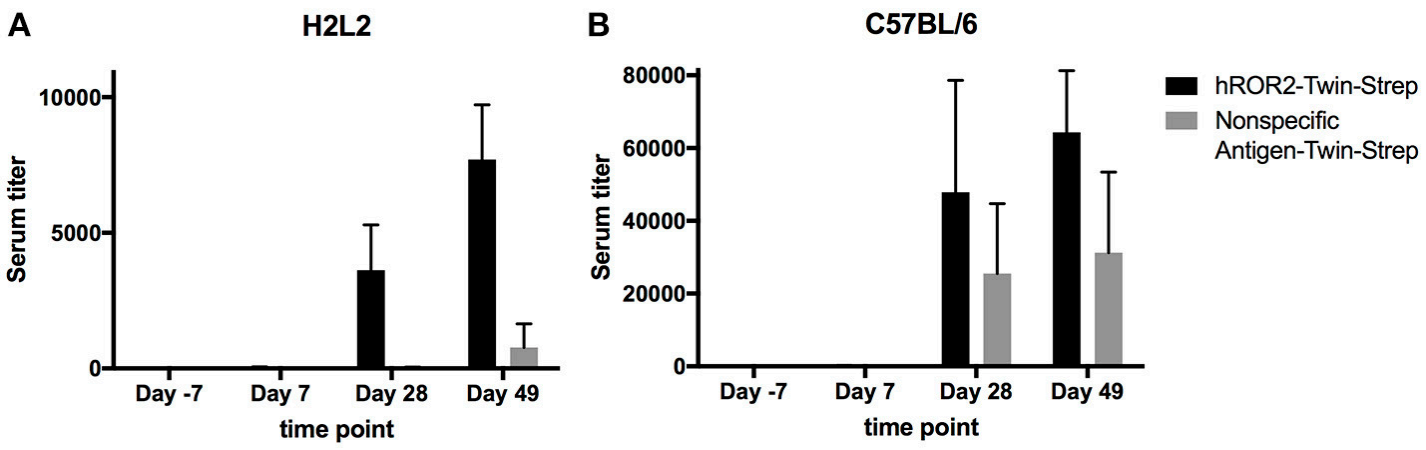
hROR2-specific serum titers in H2L2 transgenic and in C57BL/6 wild-type mice. Five H2L2 Ig transgenic mice **(A)** and 5 wild-type C57BL/6 mice **(B)** were immunized with soluble hROR2-ECD-Twin-Strep. Serum samples were collected 7 days prior to immunization as well as on days 7, 28, and 49 post-immunization, i.e., always 7 days following each immunization. Titers in serum against hROR2-ECD-Twin-Strep as well as an unrelated soluble antigen also containing the same Twin-Strep-tag (Nonspecific Antigen-Twin-Strep) to assess tag-related responses were analyzed by ELISA. Bar graphs show mean values with standard deviation indicated.

In order to generate novel monoclonal antibodies specific for human ROR2, five Ig transgenic H2L2 mice and five C57BL/6 wild-type mice (the latter as controls) were immunized with hROR2-ECD with a Twin-Strep-tag by one primary and two boost immunizations as described in the Materials and Methods. The Twin-Strep-tag appended to the C-terminus of the hROR2-ECD had been used for Strep-Tactin-mediated affinity purification of the recombinant hROR2-ECD-protein expressed in human HEK293T cells. Ig-transgenic H2L2 mice harbor small transgenic immunoglobulin gene loci with a limited set of human V, D, and J gene segments for V(D)J recombination with rat immunoglobulin constant regions on a homozygous knock-out background preventing expression of endogenous immunoglobulin heavy and kappa-light chain components. Immunization of C57BL/6 wild-type mice was included in order to benchmark the anti-hROR2-ECD-Twin-Strep humoral immune response in H2L2 Ig transgenic mice against that of wild-type mice. In the H2L2 Ig transgenic mice, serum titers against hROR2-ECD-Twin-Strep protein after primary and two boost immunizations were monitored by ELISA (Figure 2A). After primary immunization hardly any antibody titer specific for hROR2-ECD-Twin-Strep was detectable in the H2L2 Ig transgenic mice. Only after secondary (1st boost) and tertiary (2nd boost) immunization, antibody titers against the hROR2-ECD-Twin-Strep protein became detectable, reaching a mean level of approximately 1:7,700 (Standard deviation (SD): 1:2,011, *n* = 5; individual values: mouse 1357: titer 1:9,911; mouse 1359: titer 1:9,323; mouse 1363: titer 1:8,002; mouse 1358: 1:5,926; mouse 1364: 1:5,374) on day 49, after the 3rd (2nd boost) immunization, indicating a successful immunization strategy. To assess whether the immunization generated titers against the Twin-Strep-tag, serum titers against a control antigen, unrelated to hROR2-ECD, but also containing the same tag were monitored. Significantly, titers against the Twin-Strep-tag were only observed following three immunizations on day 49 and were low (Mean: 1:779, *SD*: 1:863, *n* = 5) in comparison to the hROR2-ECD-Twin-Strep-specific titers, indicating that only roughly 10% of hROR2-ECD-Twin-Strep-responses were specific for the tag. For benchmarking this immune response, five wild-type mice (C57BL/6) were also immunized in parallel with the identical immunization strategy and sampling schedule and serum titers of the different time points were assessed by ELISA as mentioned above. In wild-type mice, anti-hROR2-ECD-Twin-Strep-tag antibody titers followed the same pattern as in the H2L2 Ig transgenic mice, i.e., hardly detectable IgG anti-hROR2-ECD-Twin-Strep response after the first immunization, with increasing antigen-specific anti-IgG responses after 1st and 2nd boost immunization, but reaching higher levels of a mean of ca. 1:64,000 (*SD* 1:16,911, *n* = 5) on day 49 following three immunizations (Figure 2B). Therefore, the antigen-specific serum titers against hROR2-ECD-Twin-Strep protein in C57BL/6 mice were about 9-fold higher than those observed in H2L2 mice. Significantly, the serum titers against the Twin-Strep portion of the recombinant hROR2-ECD-Twin-Strep protein were disproportionately higher than those in H2L2 mice (mean: ca. 1:31,000, *SD*: 1:22,159, *n* = 5), indicating that in C57BL/6 mice roughly 50% of the antibody response was directed against the tag. Taken together, the immunizations in H2L2 Ig transgenic mice generated a hROR2-specific response which, although lower than in wild-type mice, was deemed sufficient for further antibody isolation. The three H2L2 Ig transgenic mice showing the highest IgG antibody serum titers after the 3rd immunization on day 49 (mice 1357, 1359, and 1363) were selected for isolation of hROR2-ECD-Twin-Strep-specific antibodies via V_H_ and V_L_ library cloning from spleen cells collected on day 49 of the experiment.

### Isolation of ROR2-binders from cellular libraries

In a first step, B cell enriched splenocytes of the three H2L2 Ig transgenic mice with the highest serum titers were stained with recombinant hROR2-ECD-Twin-Strep in order to enrich antigen-reactive splenic B lymphocytes by MACS. From the hROR2-ECD-Twin-Strep MACS enriched splenic B cells, V_H_ coding regions were amplified by RT-PCR using V_H_ forward primers and either Fcγ-specific or Fcμ-specific reverse primers for the generation of IgG and IgM derived V_H_ libraries. V_L_ coding regions were amplified using V_L_ (kappa) forward primers and Igκ light chain constant region-specific reverse primers for the generation of V_L_ libraries. The V_H_ amplicons (IgG and IgM derived) were then cloned into transposable expression vectors in frame with the coding region for human IgG_1_ heavy chain constant domains. The V_L_ amplicons were cloned into transposable expression vectors in frame with the coding region for Igκ light chain constant domains. This resulted in libraries of expression vectors for fully human IgG_1_ heavy chains (if IgG derived, designated HCγ library, and if IgM derived, designated HCμ library) and for fully human Igκ light chains (designated LCκ library), which were generated individually from each mouse. A sample of 10–15 individual plasmid clones from each library were analyzed by DNA sequencing and IgBLAST analysis to assess the quality of the transposable expression vector libraries, which confirmed that the majority of expression vectors contained *bona fide* V_H_ (>80% of analyzed plasmid clones) and V_L_ (>58% of analyzed plasmid clones) coding regions (Figure S2). This sequence analysis also confirmed that H2L2 mice indeed generated an antibody response involving the transgenic Ig heavy and light chain gene loci with fully human V_H_ and V_L_ coding regions. In addition, plasmid clones derived from HCμ libraries showed higher sequence identity to their closest human germline V gene sequence than those derived from HCγ libraries, reflecting the expected lower frequency of somatic hypermutations in IgM compared to IgG antibodies (Figure S2).

**Figure 3:**
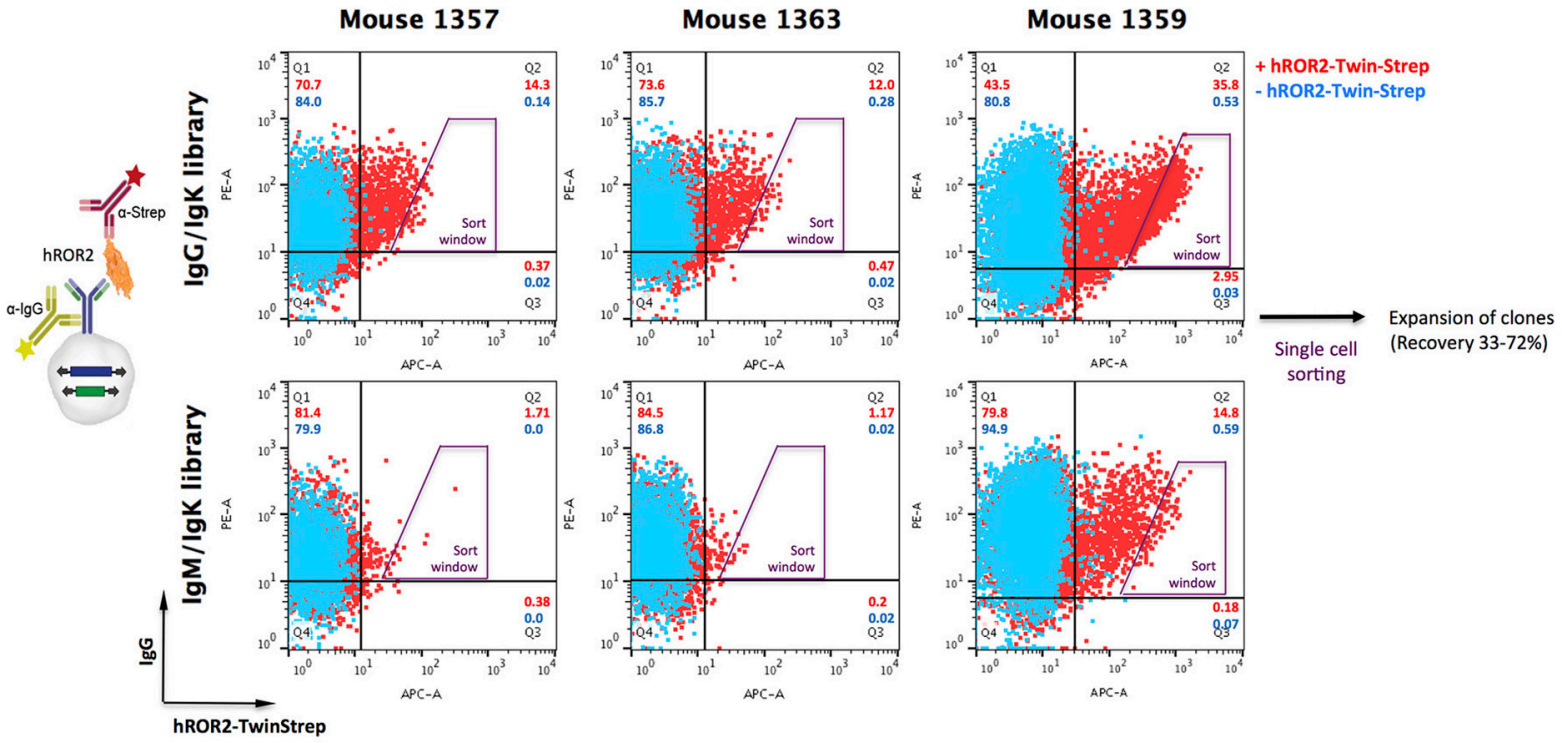
Isolation of hROR2-binders from cellular libraries by FACS. Transpo-mAb-expression libraries were constructed by amplification of coding regions for human V_H_ and V_L_ domains with human variable domain specific primers using cDNA isolated from hROR2-ECD-Twin-Strep-specific splenocytes and cloning into transposable vectors, thereby fusing the variable domains in frame to the coding regions of either HCγ1 or LCκ constant domains. By use of Cγ1 or Cμ reverse primers, V_H_ domain encoding libraries have been isolated from IgG or IgM expressing B lymphocytes. Following transposition of LCκ libraries paired with HCγ libraries (with V_H_ domains from either IgG or IgM repertoires) into L11 progenitor B cells, these cellular libraries were screened for surface IgG expression and antigen-binding by simultaneous staining with an anti-human IgG-PE and hROR2-ECD-Twin-Strep detected by an anti-Strep-mAb coupled to a fluorochrome, respectively (+ hROR2-Twin-Strep). A sample lacking hROR2-ECD-Twin-Strep was stained for comparison (– hROR2-Twin-Strep). Overlays of representative plots of 10,000 cells are shown. Per library, 288 clones with high hROR2-binding relative to IgG surface levels were single-cell sorted using the indicated sort window and expanded.

Following successful quality control of the transposable IgG_1_ heavy chain and Ig kappa light chain expression libraries, cellular libraries were created by stably transposing the LCκ-library together with either the HCγ- or the HCμ-library derived from the same mouse into L11 progenitor B cells capable of efficiently expressing IgG_1_/κL antibodies as membrane-bound IgG as well as secreted IgG. This was achieved by co-electroporating the HC and LC expression libraries with a *Piggybac* transposase expression vector (36). As described previously, the three plasmids (HC-Library:LC-library:transposase vector) were electroporated at a ratio, at which roughly 50% of transfected cells contain only a single HC and LC integration, leading to expression of one mAb per cell clone (36). Following antibiotic selection with hygromycin B and puromycin to select for clones expressing both, HC and LC, cellular libraries were stained for surface IgG expression and simultaneous binding to hROR2-ECD-Twin-Strep by FACS (Figure 3). In general, the majority of cellular library clones expressed surface IgG at varying levels, indicating a successful transposition and selection. More importantly, a fraction of these IgG-expressing cells showed variable binding to hROR2-ECD-Twin-Strep, with a correlation of cells expressing higher IgG levels also showing higher hROR2-ECD-Twin-Strep staining levels, detectable as a cell population in the upper-right quadrant of the FACS dot plots (Figure 3). From all three mice, libraries exhibiting high percentages of IgG-positive, hROR2-ECD-Twin-Strep-reactive cells were observed in the HCγ/LCκ-paired libraries (mouse 1357: 14.3%; mouse 1359: 35.8%, and mouse 1363: 12.0%) (Figure 3). In comparison to the HCγ/LCκ-libraries, HCμ/LCκ-paired libraries showed lower percentages of IgG-expressing, hROR2-ECD-Twin-Strep-reactive cells in 2 out of 3 mice (mouse 1357: 1.71%, mouse 1359: 14.8%, mouse: 1363 1.17%). This is most likely a reflection of the higher expected occurrence of antigen binders among secondary and class-switched IgG antibodies derived from a boost response, as compared to IgM antibodies, which are either derived from a primary immune response, or potentially represent antibodies from T-cell independent re-stimulated non-class-switched B lymphocytes.

Based on the obtained IgG-hROR2-ECD-Twin-Strep double stainings, a trapezoid FACS sort window was set (see Figure 3), in order to sort cells with highest hROR2-ECD-Twin-Strep-reactivity and reasonable IgG expression levels. From each library, a total of 288 clones were single-cell sorted. Recovery following sorting was high, and at least 33% of clones isolated grew out as individual cell clones (mouse 1357: HCγ/LCκ 62%, HCμ/LCκ 41%; mouse 1359: HCγ/LCκ 69%, HCμ/LCκ 72%; mouse 1363: HCγ/LCκ 44%, HCμ/LCκ 33%, Figure S3). All recovered individual cell clones obtained from the cellular libraries were selected for further binding and functional screening.

### Identification of transpo-mAb cell clones producing mAbs with hROR2-reactivity and high cell killing potency by direct functional ADC screening

**Figure 4:**
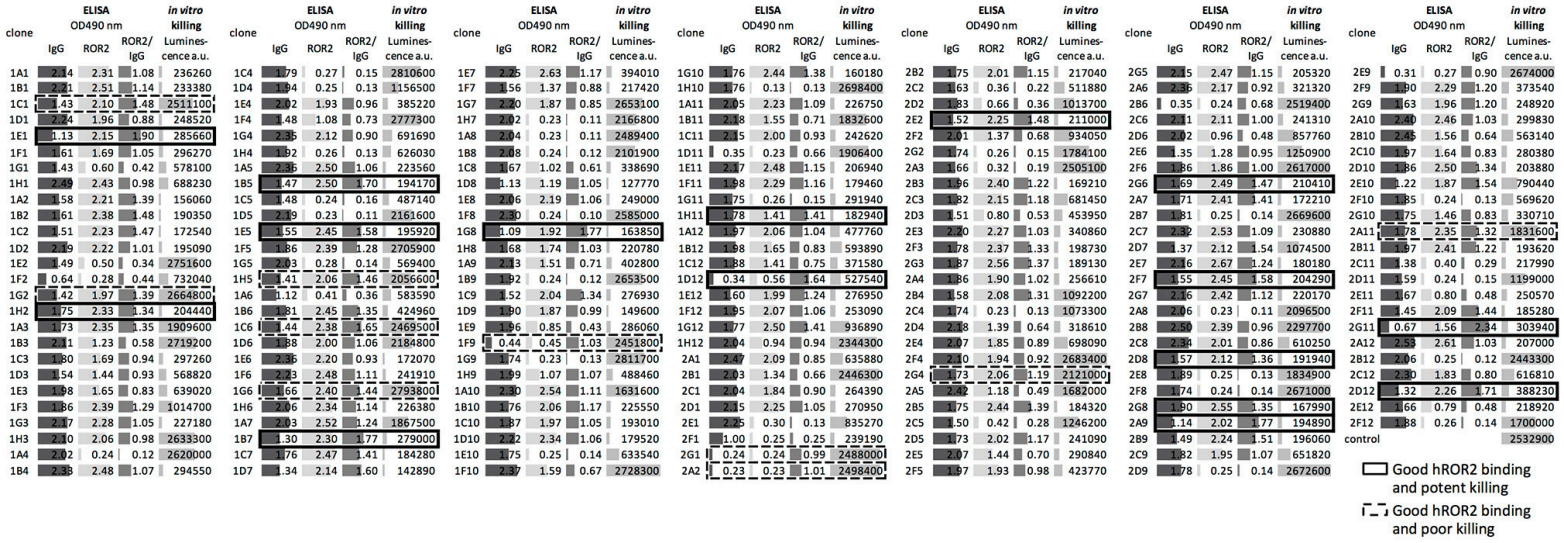
Direct screening of supernatants from sorted clones for identification of hROR2-binding clones with high potency as an ADC. Supernatants from single-cell sorted clones were screened for hROR2 binding and additionally for potency in a secondary ADC cell killing assay. Therefore, IgG levels (dark gray bars) and binding to hROR2-ECD-Twin-Strep were measured by ELISA (lightest gray bars), presented as ratios of OD490nm hROR2-ECD-Twin-Strep-binding / IgG levels (medium gray bars). Additionally, the supernatants were used to assess potency of the antibodies for cell killing in a secondary ADC assay. For this, EMT6-hROR2 cells were incubated with a 5-fold dilution of clonal supernatant for 30 min. Next, an anti-human Fcγ coupled via a cleavable linker to PNU-159682, a potent cytotoxic small molecule, was added and incubated with the cells and clonal supernatant for 3 days. EMT6-hROR2 cells incubated with only secondary ADC, but no clonal supernatant served as a control. Viable cells were then quantified using a luminescence-based cell viability assay. Lower luminescence values [arbitrary units (a.u.)] indicate more potent killing (light gray bars). The IgG_1_/Igκ library of mouse 1357 is shown as a representative. Examples of clones with strong hROR2 binding and potent (solid cell border) as well as poor *in vitro* killing (dashed lines) are indicated.

In the next step, all recovered individual cell clones were evaluated for their binding to hROR2-ECD-Twin-Strep, as well as for their activity in hROR2-specific cell killing using cells overexpressing hROR2 protein on the cell surface and a toxin-conjugated anti-IgG secondary reagent. To assess binding to hROR2-ECD-Twin-Strep, supernatants from sorted clones containing secreted antibodies were assessed for IgG expression and binding to hROR2-ECD-Twin-Strep by ELISA in a single-well measurement. Then, the ratio between OD = 490 nm (optical density at a wavelength of 490 nm) for hROR2-ECD-Twin-Strep-binding and OD = 490 nm for IgG expression was calculated to normalize hROR2-ECD-Twin-Strep-binding to antibody expression (Figure 4–medium gray bars). In the HCγ/LCκ libraries, a large number of clonal supernatants showed high hROR2-ECD-Twin-Strep-binding/IgG-expression ratios, indicating that the majority of the single-cell sorted clones expressed antibodies with hROR2-ECD-Twin-Strep-reactivity (Figure 4, Figure S4A). In contrast, fewer clones with high hROR2-ECD-Twin-Strep-binding/IgG-expression ratios were observed in the HCμ/LCκ-libraries (Figure S4A), demonstrating a lower prevalence of strong hROR2-ECD-Twin-Strep-binders in these libraries that were derived from non-class-switched B lymphocytes.

In addition to the measurement of hROR2-ECD-Twin-Strep-binding by ELISA, the supernatants were assessed for *in vitro* cell killing potency in a secondary ADC assay. In this approach, clonal supernatants containing secreted antibodies were incubated with EMT6 cells overexpressing hROR2, followed by addition of an anti-human Fcγ IgG coupled to the potent cytotoxic anthracycline PNU-159682 via a cleavable linker, as described in the Materials and Methods. Viable cells were quantified following a 3 days incubation using a luminescence-based cell viability assay in a single-well measurement (Figure 4–light gray bars). In all libraries, luminescence signals ranging from low to high were observed, thus representing clonal supernatants with potent to poor *in vitro* cytotoxicity, respectively (Figure 4, Figure S4B). More importantly, the majority of cell clones exhibited both, strong hROR2-ECD-Twin-Strep-binding as assessed by ELISA, as well as high potency toward hROR2-expressing EMT6 cells by a secondary ADC *in vitro* cell killing assay, indicating suitability of the antibodies expressed by these clones as ADCs (Figure 4–solid border). Interestingly, several clones were identified that showed strong binding to hROR2-ECD-Twin-Strep in ELISA, but only poor cytotoxicity in the secondary ADC assay, indicating that not every hROR2-ECD-Twin-Strep-specific antibody is capable of mediating effective delivery of the cytotoxic payload of the secondary ADC to the hROR2-positive EMT6 cells (Figure 4–dashed line). This demonstrates that our functional assay is capable of distinguishing hROR2-specific antibodies with and without *in vitro* cell killing activity in a secondary ADC assay. While many clonal supernatants that induced strong *in vitro* cell killing were observed in the HCγ/LCκ libraries, there were only few potent mAbs in the HCμ/LCκ libraries (Figure S4B). This matches the lower occurrence of strong hROR2-binders in these libraries. To confirm their *in vitro* cell killing potency, IgG levels of selected clonal supernatants were quantified and secondary ADC assays were then carried out using serial dilutions of clonal supernatants at defined antibody concentrations. This analysis confirmed the relative cell killing potencies of the selected clones (Figure S5). These results suggest that our functional screening not only allows the straightforward identification of antibodies that strongly bind hROR2, but also to rapidly differentiate between antigen-binding clones with or without activity in an *in vitro* ADC cell killing assay. Based on the antigen-reactivity, IgG expression and cell killing activity, 22 hROR2-reactive clones were selected for antibody sequence recovery. From these 22 clones, six clones each originated in mice 1357 and 1363, and 10 clones originated in mouse 1359.

### Recovery of V_H_ and V_L_ coding regions from selected hROR2-reactive transpo-mAb cell clones and characterization of novel fully human anti-hROR2 antibodies

**Figure 5:**
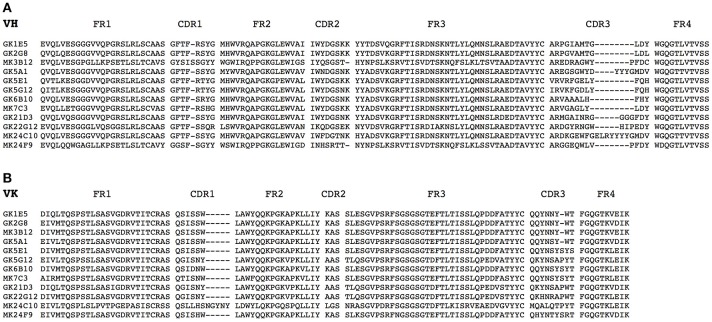
Alignment of amino acid sequences of identified hROR2-specific antibodies. Heavy chain and Kappa light chain variable regions were PCR amplified from single-cell L11 clones by reverse transcription and subjected to Sanger sequencing. The derived amino acid sequences of recovered V_H_ **(A)** and V_κ_ **(B)** are shown. Antibodies were annotated using IgBLAST. FR, framework region; CDR, complementarity-determining region. Dashes indicate gaps due to the alignment.

**Figure 6:**
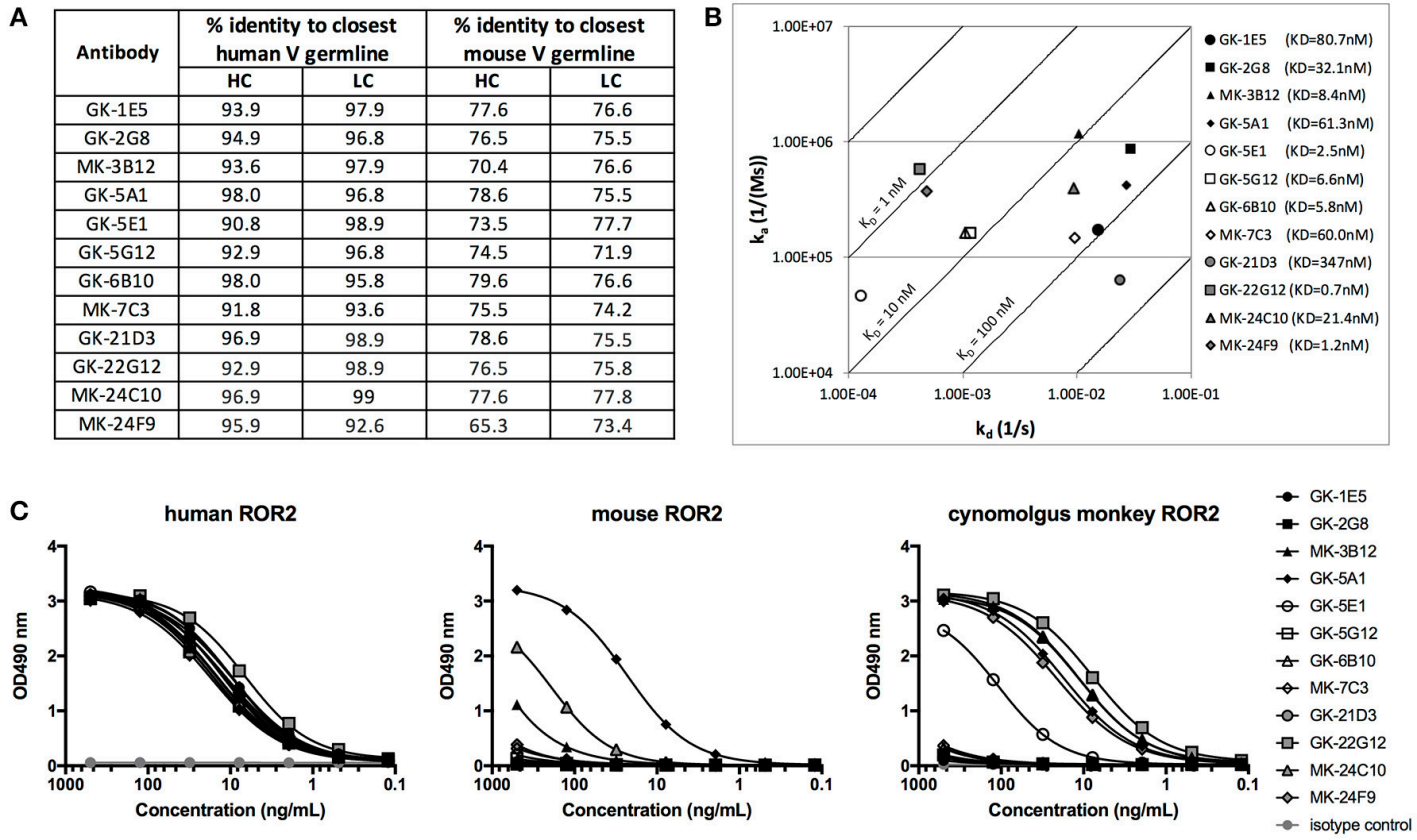
Characterization of novel hROR2-specific antibodies. **(A)** Identity to the closest human and mouse germline V gene sequence for heavy (HC) and light chain (LC) as a measure of “humanness” was evaluated *in silico* using IgBLAST. **(B)** Isoaffinity plot showing association (k_a_) and dissociation constants (k_d_) as determined by surface plasmon resonance (SPR) using clonal supernatants. Diagonal lines represent equal affinities. **(C)** Purified antibodies were assessed for binding to soluble human, cynomolgus monkey and mouse ROR2-ECD-Twin-Strep by ELISA.

In the next step, V_H_ and V_L_ coding regions from 22 strongly hROR2-ECD-Twin-Strep-reactive cell clones that also showed potent *in vitro* cell killing activity in the functional ADC assay were recovered by RT-PCR from the stably Ig heavy chain and Ig light chain transposed cell clones, and cloned into an EBNA-based expression vector and sequenced (Figure 5). These clones displayed 12 distinct clonotypes as defined by their heavy chain CDR3 sequences with clonotypes GK-1E5, GK-2G8, and MK-3B12 originating in mouse 1357; clonotypes GK-5A1, GK-5E1, GK-5G12, GK-6B10, and MK-7C3 originating in mouse 1363; and clonotypes GK-21D3, GK-22G12, MK-24C10, and MK-24F9 originating in mouse 1359. While most of the corresponding light chain CDR3 sequences were also distinct, clones GK-1E5, GK-2G8 and MK-3B12, as well as GK-6B10 and MK-7C3, shared the same light chain CDR3 sequences, respectively. It was confirmed that all recovered sequences indeed represented fully human antibodies, as they shared a higher degree of sequence identity to their closest human than to their closest mouse germline V gene sequence, as assessed by IgBLAST. For the HC sequences, identities to the closest human or mouse germline V gene sequence ranged from 90.8–98 to 65.3–79.6%, respectively (Figure 6A). For the LC sequences, identities to the closest human or mouse germline V gene sequence ranged from 92.6–99 to 71.9–77.8%, respectively. This *in silico* IgBLAST analysis also revealed the germline V gene usage of the identified anti-hROR2-clonotypes (Table S1). In the HC sequences, the majority of clonotypes used the V_H_3-33 gene segment (*n* = 9), while one clonotype each used the V_H_4-38, V_H_3-7 or V_H_4-34 gene segment. In the LC sequences, the most prevalent Vκ gene segment was Vκ1-5 (*n* = 8), followed by Vκ1-27 (*n* = 3) and Vκ2-28 (*n* = 1). These identified V gene sequences are in line with frequently used human V gene sequences (40).

The 12 distinct clonotypes defined above were expressed at a larger scale in mammalian HEK293T cells and purified using Protein A. To assess the functional properties of the isolated, now recombinantly expressed monoclonal antibodies, affinity to hROR2-ECD-Twin-Strep was measured by Surface Plasmon Resonance (SPR) (Figure 6B, Figure S6). The SPR measurements confirmed that all recovered and recombinantly expressed antibodies showed hROR2-ECD-Twin-Strep binding by SPR with affinities ranging between 0.7 and 347 nM, with 6 of the 12 mAbs having affinities below 10 nM, 5 mAbs having affinities between 10 and 100 nM, and one mAb clone with 347 nM affinity. However, the clones displayed variable association and dissociation rates. 5 mAbs showed favorable and very slow dissociation rates (*e.g*. GK-22G12, MK-24F9, GK-5E1, GK-6B10, and GK-5B12), including some clones with the highest affinities.

Another important characteristic of therapeutic monoclonal antibodies is their cross-reactivity to their specific antigen in relevant toxicology species, e.g., mice and cynomolgus monkeys. Therefore, binding of the novel, fully human hROR2-specific mAbs to cynomolgus monkey as well as to mouse ROR2 was evaluated by ELISA (Figure 6C). While all tested antibodies strongly bound to human ROR2, only MK-3B12, GK-5A1, GK-5E1, MK-24C10, MK-24F9, and GK-22G12 were capable of binding cynomolgus monkey ROR2-ECD-Twin-Strep. Interestingly, GK-5A1 also strongly bound to mouse ROR2-ECD-Twin-Strep, with weaker binding observed also for MK-3B12 and MK-24F9. While the specific epitopes recognized by the identified antibodies have not yet been mapped, these results indicate that different epitopes are recognized by these different antibodies.

Taken together, the combined screening approach involving (1) immunization of human Ig-transgenic mice, (2) enrichment of antigen-reactive splenic B lymphocytes from immunized animals by MACS, and (3) concomitant screening of Transpo-mAb IgG display libraries for hROR2 binding and ADC functionality, resulted in the successful identification of a diverse panel of 12 novel, fully human anti-ROR2-specific monoclonal antibodies with nanomolar affinities to hROR2. Furthermore, some of these antibodies recognized ROR2 from relevant toxicology species.

### Generation and evaluation of hROR2-specific ADCs

**Figure 7:**
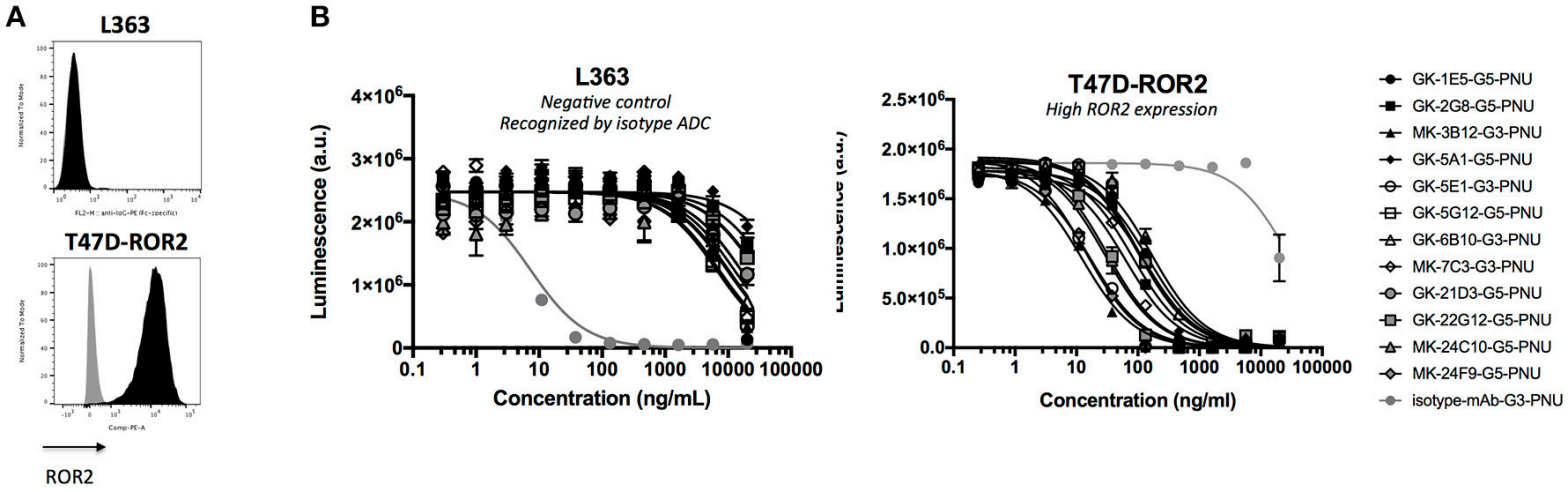
Validation of ADCs. **(A)** hROR2 surface expression in cell lines used in this study as determined by FACS using the hROR2-specific antibody XBR2-401 (37) detected using a PE-labeled anti-human Fcγ. Black histograms, ROR2 staining; gray histograms, control staining with only the secondary antibody. **(B)** Following recovery of V_H_ and V_L_ sequences by RT-PCR cloning from hROR2-ECD-Twin-Strep reactive Transpo-mAb cell clones and expression in HEK293T cells, purified recombinant antibodies were site-specifically conjugated to a derivative of the potent cytotoxic payload PNU-159682 by sortase-mediated antibody conjugation (SMAC-technology^TM^) to the C-termini of the heavy and light chain constant domains. These ADCs were tested for their cell killing activity on hROR2 negative (L363), and hROR2-high (T47D-hROR2) cell lines. Viability of the cells is plotted in arbitrary units (a.u.) of luminescence on the y-axis as a function of the concentration of the ADCs on the x-axis as a result of a CellTiter-Glo cell-killing assay. hROR2-negative L363 cells were used as negative controls (with an isotype-control mAb in light gray that binds to a TAA on L363). G3 and G5 designate the length of a glycine linker used in the ADCs, which has previously been shown not to affect ADC potency. Datapoints represent mean of two replicates and error bars represent *SD*.

Finally, the activity of the 12 novel hROR2-specific monoclonal antibodies for *in vitro* cell killing was determined after conjugation of the recombinant mAbs to a derivative of the strong cellular toxin PNU-159682. For this, ADCs were generated by site-specific conjugation of a Gly_3_-EDA-PNU, or a Gly_5_-EDA-PNU linker toxin to the C-termini of the Ig heavy and light chains by SMAC-technology^TM^ conjugation as described previously (39). The cell killing activity of these ADCs was then assessed on highly hROR2 expressing breast cancer cells T47D-hROR2, and, as a negative control, on hROR2-negative L363 multiple myeloma cells (Figure 7A), as described in the Materials and Methods. Highly potent killing of the highly hROR2-expressing cell line T47D-hROR2 cells was observed with all hROR2-specific ADCs, with IC_50_ values ranging from 12.3 to 168 ng/ml (Table S2). In contrast, the ROR2-negative cell line L363 showed only minor cell killing at very high ADC concentrations. As a further control, a L363-reactive ADC (designated: isotype-mAb-G3-PNU) was included in the panel of ADCs tested for cell killing on the hROR2-negative and hROR2-positive cells, demonstrating that L363 cells, but not T47D-hROR2 cells could be killed with a L363-reactive ADC. Taken together, these data clearly demonstrate that the novel, fully human anti-hROR2 mAbs have favorable properties as ADCs for the specific targeting of hROR2 expressing cells. Interestingly, the ADC with the lowest cell killing activity (based on mAb GK-6B10) was among the higher affinity ADCs (K_D_ of 5.8 nM). Conversely, the lowest affinity ADC (based on mAb GK-21D3 with a K_D_ of 347 nM) had comparable cell killing activity as other, higher affinity ADCs, where the mAbs displayed single-digit nanomolar affinities.

## Discussion

ADCs represent a promising therapeutic principle for cancer treatment, because cellular toxins can more specifically be targeted to tumor cells via TAA-specific antibodies. A potent ADC kills tumor cells in a target-dependent manner by specifically binding a TAA via its antibody moiety, which at the same time needs to induce efficient internalization of the formed antigen-ADC complex for efficient delivery of the cytotoxic payload into the tumor cell to release its cytotoxic potential. Because antibodies do not all internalize at the same rate (26, 27), ADC discovery would greatly benefit from functional screening for efficient internalization and eventually cell killing activity during the very early stages of antibody discovery, thereby reducing the time and costs for identification of suitable mAbs for ADC strategies and for pre-clinical development.

Here, we incorporated the functional screening for potent ADCs into the discovery process for novel, fully human hROR2-specific antibodies by combining immunization of transgenic mice with the Transpo-mAb display platform (36). Antigen binding capability and ADC potency with a secondary ADC killing assay was simultaneously evaluated by screening cell culture supernatants from single-cell sorted Transpo-mAb clones with an anti-human Fcγ antibody coupled to a derivative of the cytotoxic payload PNU-159682 via a Cathepsin-cleavable linker (41, 42). This secondary ADC reagent enters the cell by “piggybacking” onto the internalizing antibody, thereby allowing high-throughput screening for internalizing antibodies without prior cloning, re-expression of mAbs, and generation of ADCs from selected cell clones. Strategies for screening for internalizing antibodies using a secondary antibody to deliver a toxic payload have been described previously, including delivery of a protein toxin (43) or a small molecule cytotoxic agent such as Monomethyl auristatin E (MMAE) (44). However, it is important to note that these approaches employed hybridoma supernatants for functional screening, and that the recombinant cloning and sequence determination of hybridoma mAbs is often associated with significant challenges. In contrast, the approach described here involves recombinant libraries where the sequences of identified antibodies are readily available. In addition to the delivery of toxic payloads, other strategies to functionally screen for internalizing antibodies using secondary reagents have been described, e.g., using a pH-sensitive dye such as CypHer5E (45) or a dual label consisting of the fluorophore DL650 and the quencher DL650-QC1 (46). Similar to these assays, our secondary ADC assay likely requires internalization of the antigen-secondary ADC complex by endocytosis and delivery of the complex to the lysosome for proper release and function of the payload in the low pH, protease-rich environment (44, 47). Thus, while both reported fluorescence-based high-throughput methods allow measuring antibody internalization, our assay more closely mimics a primary ADC as it contains a cytotoxic payload and is therefore more predictive of its function and potency as a directly conjugated ADC.

It is important to note that our assay uses an IgG-based rather than a Fab-based secondary reagent. Through the bivalency of the IgG, there is a potential risk for crosslinking of the antigen which could result in an enhanced internalization rate (48). However, these differences are expected to be minor, and it has been found that incubation of the naked antibody Herceptin with either Fab- or IgG-based secondary ADC reagents on HER2-expressing cell lines resulted in comparable IC_50_ values (*personal communication from Moradec Inc.*, www.moradec.com). While we used PNU-159682 as a model toxin due to its high potency (38, 49), the assay described herein is versatile and can be used with secondary ADC reagents conjugated to any other toxin, including tubulin-inhibitors, and using any suitable target-positive cell line.

In many library screening approaches, antibodies are screened for binding to their target, e.g., by ELISA. However, there is evidence that high affinity does not always correlate with good internalization (50). Similarly, we observed that affinity measured by SPR was not directly correlated with potency as an ADC. While some antibodies with low nanomolar K_D_ values induced potent cytotoxicity, we also observed that an ADC based on clone GK-6B10 (KD = 5.8 nM) mediated the lowest potency on T47D-hROR2 cells, whereas an ADC based on the low affinity clone GK-21D3 (347 nM) was quite potent (Figures 6B, 7). These differences are likely to be related to the recognition of different epitopes, which is supported by the observation that 4 of the 5 cynomolgus monkey ROR2-crossreactive mAbs (MK-3B12, GK-22G12, MK-24C10, MK-24F9) give rise to ADCs ranking among the 5 most potent ADCs. This underlines that internalization is a complex process not only depending on binding affinity to the cognate target, but also on the epitope the antibody recognizes, the internalization rate of the antigen-ADC complex and the target surface expression (26, 51). This further highlights the importance of functionally screening antibodies for their ability to deliver a cytotoxic payload, rather than just for affinity, in a high-throughput setting during early discovery of ADCs.

One important aspect of our present study is the use of fully human immune libraries in the functional screening for ADC candidates. The discovery of fully human antibodies is highly desired for therapeutic use due to an expected lower risk of immunogenicity associated with a better efficacy and safety profile. Human antibodies can be obtained by numerous methods, including screening phage or yeast display libraries, identification of human mAbs from human PBMCs or antibody discovery from various human Ig transgenic mice (52–56). While some human antibody transgenic mouse strains achieve comparable serum titers to wild-type mice in response to antigen challenge, most transgenic mice have lower titers than wild-type mice (52, 57). We also observed lower serum titers in H2L2 Ig transgenic mice compared to wild-type mice following the same immunization strategy. Importantly, the serum titers in H2L2 mice we observed were in line with previous reports (Patent WO2017063593A1, WO2017016497A1), where titers around 1:10,000 as measured by ELISA were achieved using different antigens for immunization. This is possibly explained by the fact that the H2L2 transgenic mice harbor a small subset of variable, diversity and joining gene segments in comparison to the endogenous germline repertoire of variable, diversity and joining gene segments in the wild-type mouse genome. However, it is also possible that the human V_H_ and V_L_ coding regions assembled from the transgenic miniloci in combination with the rat constant regions contained in these transgenic constructs may not be able to generate the same signal quality during early B lymphopoiesis in the bone marrow of the transgenic mice, which may result in lower peripheral B cell numbers. During early pre-B cell development in the bone marrow of wild-type mice, V(D)J recombination on the heavy chain locus precedes V(D)J recombination on the light chain locus (58) and pre-B cells with a productive heavy chain rearrangement are expanded by expression of a pre-B cell receptor (pre-BCR) formed by the μH chain with the VpreB and λ_5_ surrogate light chain components (59, 60). This proliferative expansion of so-called pre-B-II cells with a signaling pre-BCR is critical for an effective generation of a full peripheral B cell compartment, as evidenced by mice with lack of either surrogate light chain components (61), or lack of the μH chain transmembrane anchor (62), or of the pre-BCR/BCR signaling components, B29 and mb-1. In addition, it is also possible that the human V_H_ and V_L_ regions following productive rearrangements do not mediate proper association with the murine surrogate light chain components, VpreB and λ_5_, thus compromising pre-BCR signaling and generation of a proliferating preB-II cell compartment. Equally, it is possible that the transgenic heavy and light chain gene loci do not mediate the proper sequential V(D)J recombination events on the heavy and the light chain gene loci and that IgMs from simultaneously rearranged transgenic heavy and light chain gene loci are expressed, which may not be able to trigger the proliferative expansion of pre-BCR expressing pre-B-II cells to the same extent as in wild-type mice. This would result in only a slow filling up of a normally sized peripheral B cell population, as observed in surrogate light chain knock-out mice (59), which depend on productive V to J gene rearrangements of conventional light chain loci, in the absence of the capacity to express a pre-BCR comprising a surrogate light chain (61). Without a detailed analysis of the pre-B and B cell subsets in H2L2 Ig transgenic mice it is not possible to predict, which of the discussed mechanisms accounts for the roughly 9-fold reduced antibody titers upon immunization of H2L2 mice vs. wild-type mice. Nevertheless, despite a weaker humoral immune response, we were able to identify a very diverse set of novel high affinity and fully-human anti-ROR2-specific antibodies from H2L2 mice for further characterization and development.

In summary, the combination of an immunization strategy of H2L2 transgenic mice with the IgG Transpo-mAb display platform expressing fully human antibodies has proven to be a powerful and effective method to identify novel hROR2 antibodies with potent cell killing activity as ADCs early during the antibody discovery process. Through the straightforward functional screening of soluble antibodies directly from the supernatants of sorted cells, we identified 12 fully human, monoclonal hROR2-specific antibodies with distinct characteristics with the potential to result in potent ADCs and the potential to be promising drug candidates for human tumor therapy. The functional screening approach presented here will be highly beneficial for the fast and efficient discovery of fully human antibodies and greatly help accelerate the development of ADCs.

## Author contributions

IH, LW, UG, and RB conceived the experiments. IH, M-CB-E, KM, and FW conducted the experiments and analyzed the data. IH, RB, and UG wrote the paper.

### Conflict of interest statement

IH, LW, M-CB-E, UG, and RB are employees of NBE-Therapeutics Ltd and hold stocks or stock options of NBE-Therapeutics Ltd. This work has been included in a patent application by NBE-Therapeutics Ltd. The remaining authors declare that the research was conducted in the absence of any commercial or financial relationships that could be construed as a potential conflict of interest.

## Supplementary material

Figure S1 Primer sequences for library construction. Primers were either adapted from ^*^(1) Sblattero and Bradbury (63); (2) Tiller et al. (64); (3) Berry et al. (65); or (4) developed in-house.

Figure S2 Quality control analysis of the transposable expression vector libraries. 10–15 individual plasmids derived from the HCγ, HCμ, and LCκ libraries of mice 1357, 1359, and 1363 were analyzed using DNA sequencing and *in silico* IgBLAST analysis. IgBLAST was used to annotate the CDR3 sequences and to determine the sequence identities of the identified variable domains to their closest human germline V gene sequence. Sequences with an early stop codon were defined as incomplete.

Figure S3 Recovered clones following single-cell sorting for hROR2-binding cellular library clones. A total of 288 clones per library were sorted and expanded. The number and percentage of recovered clones are indicated.

Figure S4 Direct screening of supernatants from sorted clones allows identification of hROR2-binding clones with high potency as an ADC. **(A)** Supernatants from sorted single-cell cellular clones of the (GK) and (MK) libraries (GK = V_H_ sequences cloned from IgGs, MK = V_H_ sequences cloned from IgMs based on different reverse primers used) derived from mice 1357, 1359, and 1363 were functionally screened for IgG levels and hROR2-ECD-Twin-Strep binding by ELISA in a single-well measurement. Normalized hROR2 binding was expressed as the ratio of OD = 490 nm hROR2-ECD-Twin-Strep binding and OD = 490 nm IgG expression for all clones from the different libraries and is shown as box-plots. **(B)** All supernatants were also assessed for potency as an ADC using a secondary ADC assay. For this, EMT6-hROR2 cells were incubated with clonal supernatant without normalization for IgG levels for 30 min, before addition of an anti-human Fcγ coupled via a cleavable linker to PNU-159682. Viable cells were quantified following a 3 days incubation using a luminescence-based cell viability assay. Lower luminescence values in the box-plots indicate more potent killing.

Figure S5 Validation of *in vitro* killing potency of selected clones from functional ADC screening. Twelve clonal L11 supernatants with potent *in vitro* killing and four supernatants with poor *in vitro* killing (GK-1C6, GK-1G6, MK-3E5, and MK-3A11) were selected for testing in a secondary ADC assay using a range of defined concentrations to confirm their *in vitro* cell killing potency. To do so, IgG levels of the supernatants were quantified by ELISA and IgG concentration in all supernatants was adjusted to the lowest expressor. EMT6-hROR2 cells were incubated with 2-fold serial dilutions of these normalized clonal supernatants for 30 min, followed by the addition of an anti-human Fcγ coupled via a cleavable linker to PNU-159682. After a 3 days incubation, viable cells were quantified using a luminescence-based cell viability assay. **(A)** Viability of the EMT6-hROR2 cells plotted in arbitrary units (a.u.) of luminescence on the y-axis as a function of the IgG concentration in the supernatants on the x-axis. Clonal supernatants that had potent or poor *in vitro* cell killing potency in the initial functional ADC screening are shown in black or gray, respectively. **(B)** shows luminescence values that had been determined in the one-well secondary ADC assay during functional ADC screening compared to the IC_50_ values which were determined in the secondary ADC assay using serial dilutions of normalized supernatants for the same clonal supernatants. IC_50_ values were calculated using a four-parameter curve fitting model in GraphPad Prism. n/a indicates IC_50_ values that could not be calculated due to a lack of *in vitro* killing.

Figure S6 SPR sensorgrams of anti-hROR2 antibodies. Affinities were measured by multi-cycle SPR on a Biacore T200 instrument (GE Healthcare). Antibodies were captured by Protein A or G immobilized on a CM5 sensor chip, followed by the addition of hROR2-ECD-Twin-Strep used as 2-fold serial dilutions ranging from 40 to 2.5 nM. KD values as a measure of binding affinity are indicated.

Table S1 Germline V gene usage of identified anti-hROR2-clonotypes. The closest human germline V gene sequences of heavy (HC) and light chain (LC) were identified *in silico* using IgBLAST.

Table S2 *In vitro* cell killing by anti-hROR2-ADCs. IC_50_ values (ng/ml) reported here represent the IC_50_ values of the hROR2-specific ADCs tested for their cell killing activity on hROR2-negative L363 and hROR2-high EMT6-hROR2 cell lines in Figure 7. IC_50_ values were calculated from the mean of two replicates using a four-parameter curve fitting model in GraphPad Prism.
