# Supplementary figures and images for: Novel Antibody Drug Conjugates Targeting Tumor-Associated Receptor Tyrosine Kinase ROR2 by Functional Screening of Fully Human Antibody Libraries Using Transpo-mAb Display on Progenitor B Cells
Source: Front Immunol. 2018 Nov 2;9:2490. doi: 10.3389/fimmu.2018.02490 (PMC6224377; doi:10.3389/fimmu.2018.02490)

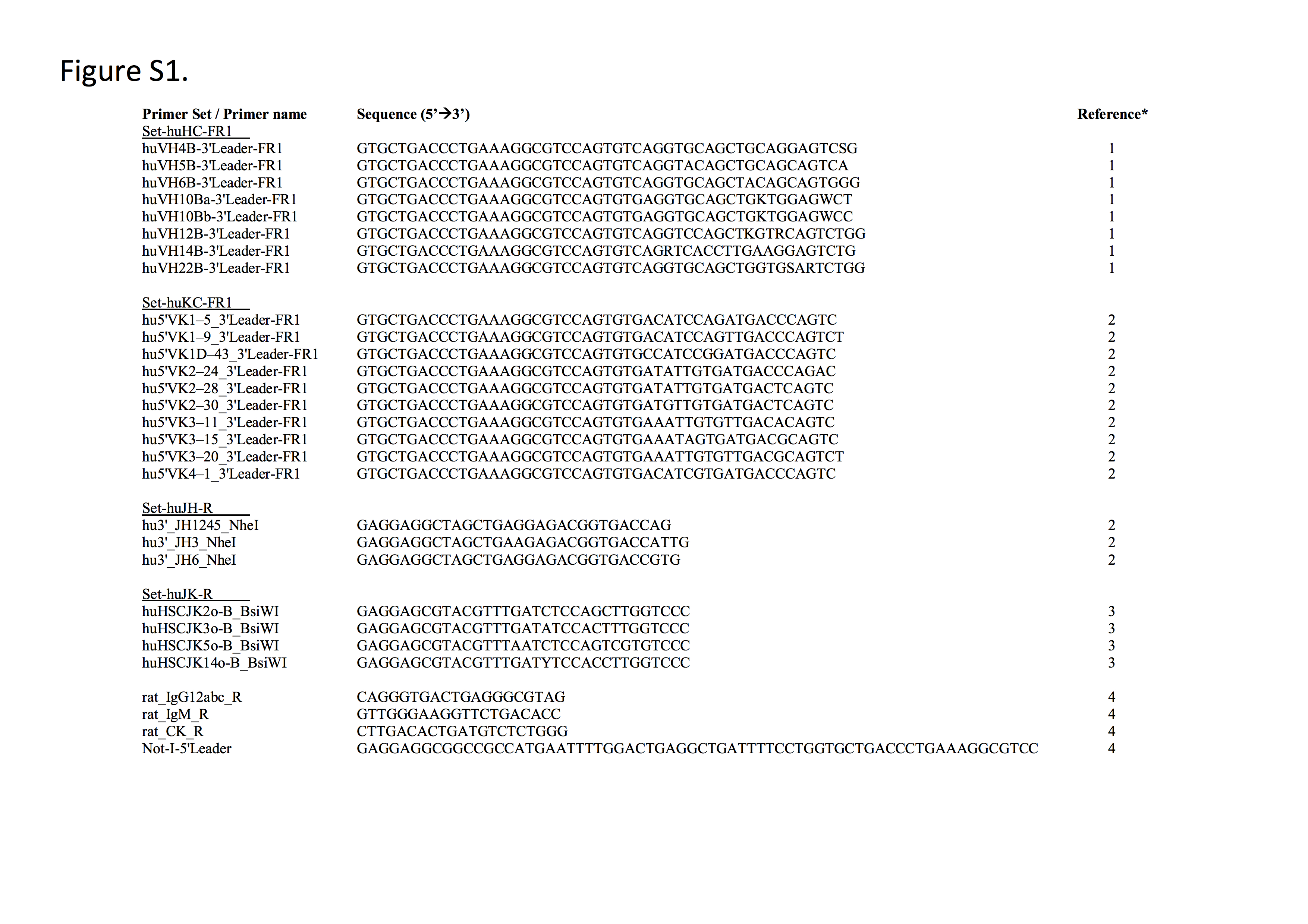

Supplement: Figure S1 — Primer sequences for library construction. Primers were either adapted from *(1) Sblattero and Bradbury (63); (2) Tiller et al. (64); (3) Berry et al. (65); or (4) developed in-house. [file Image_1.JPEG]

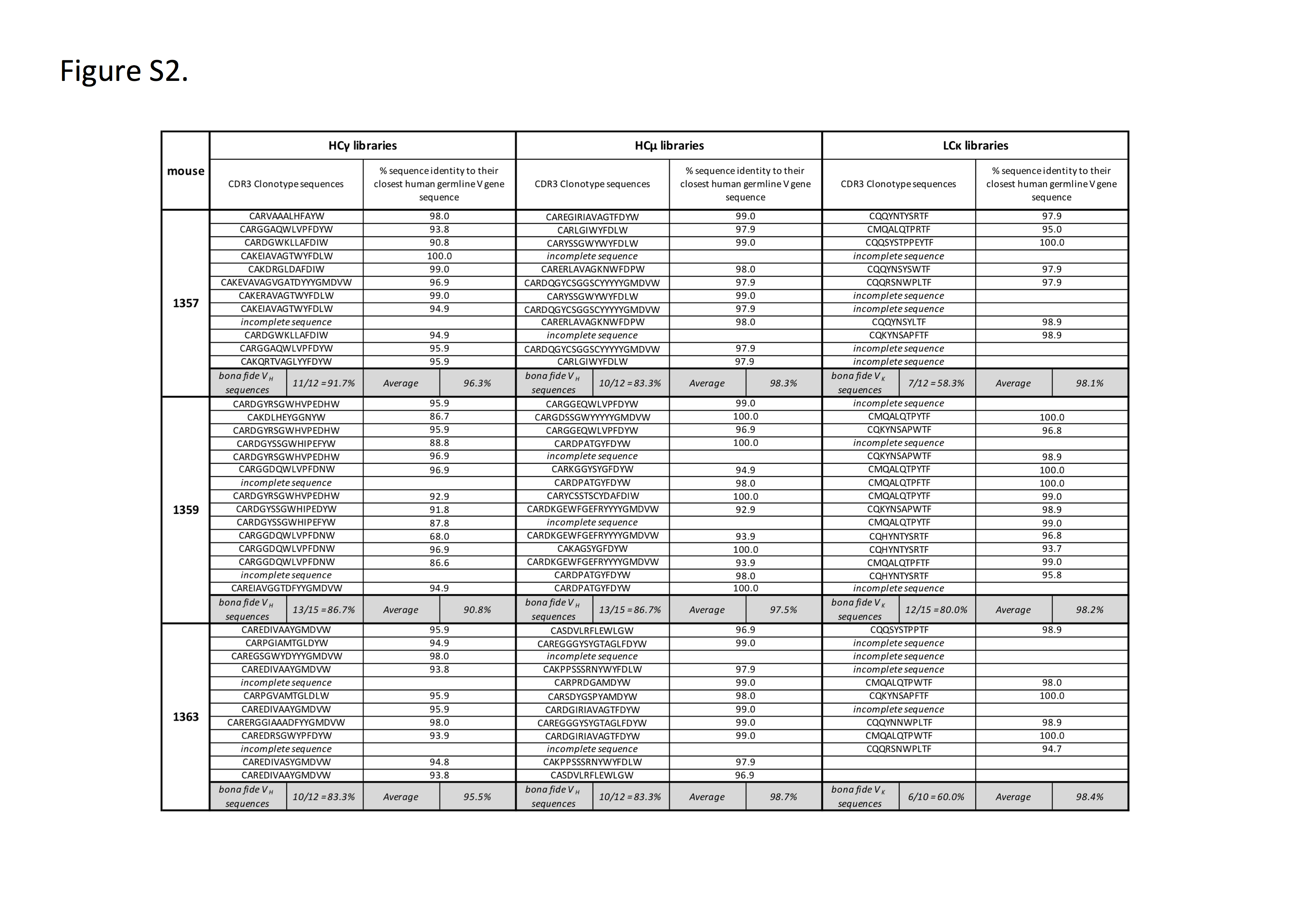

Supplement: Figure S2 — Quality control analysis of the transposable expression vector libraries. 10–15 individual plasmids derived from the HCγ, HCμ, and LCκ libraries of mice 1357, 1359, and 1363 were analyzed using DNA sequencing and in silico IgBLAST analysis. IgBLAST was used to annotate the CDR3 sequences and to determine the sequence identities of the identified variable domains to their closest human germline V gene sequence. Sequences with an early stop codon were defined as incomplete. [file Image_2.JPEG]

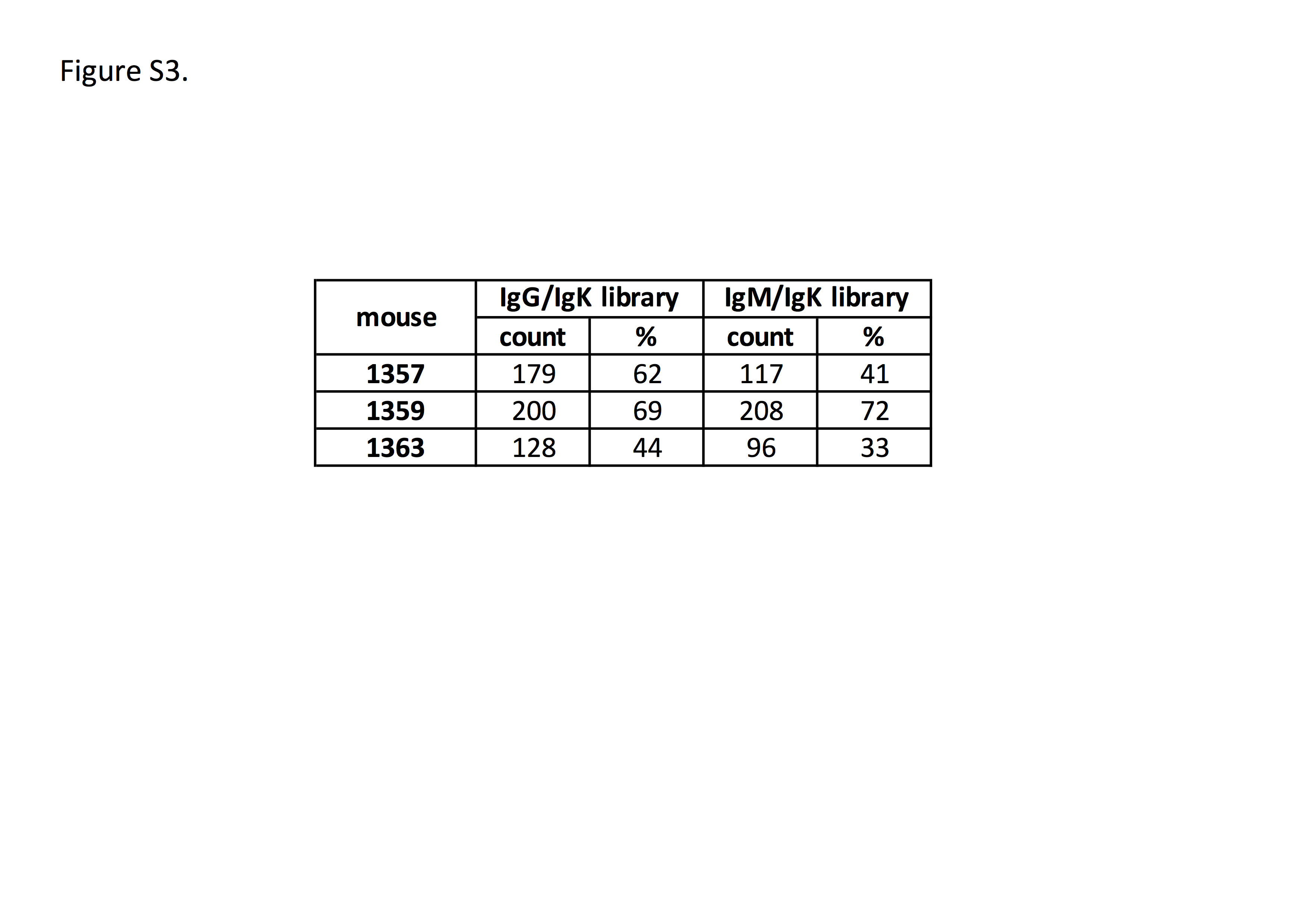

Supplement: Figure S3 — Recovered clones following single-cell sorting for hROR2-binding cellular library clones. A total of 288 clones per library were sorted and expanded. The number and percentage of recovered clones are indicated. [file Image_3.JPEG]

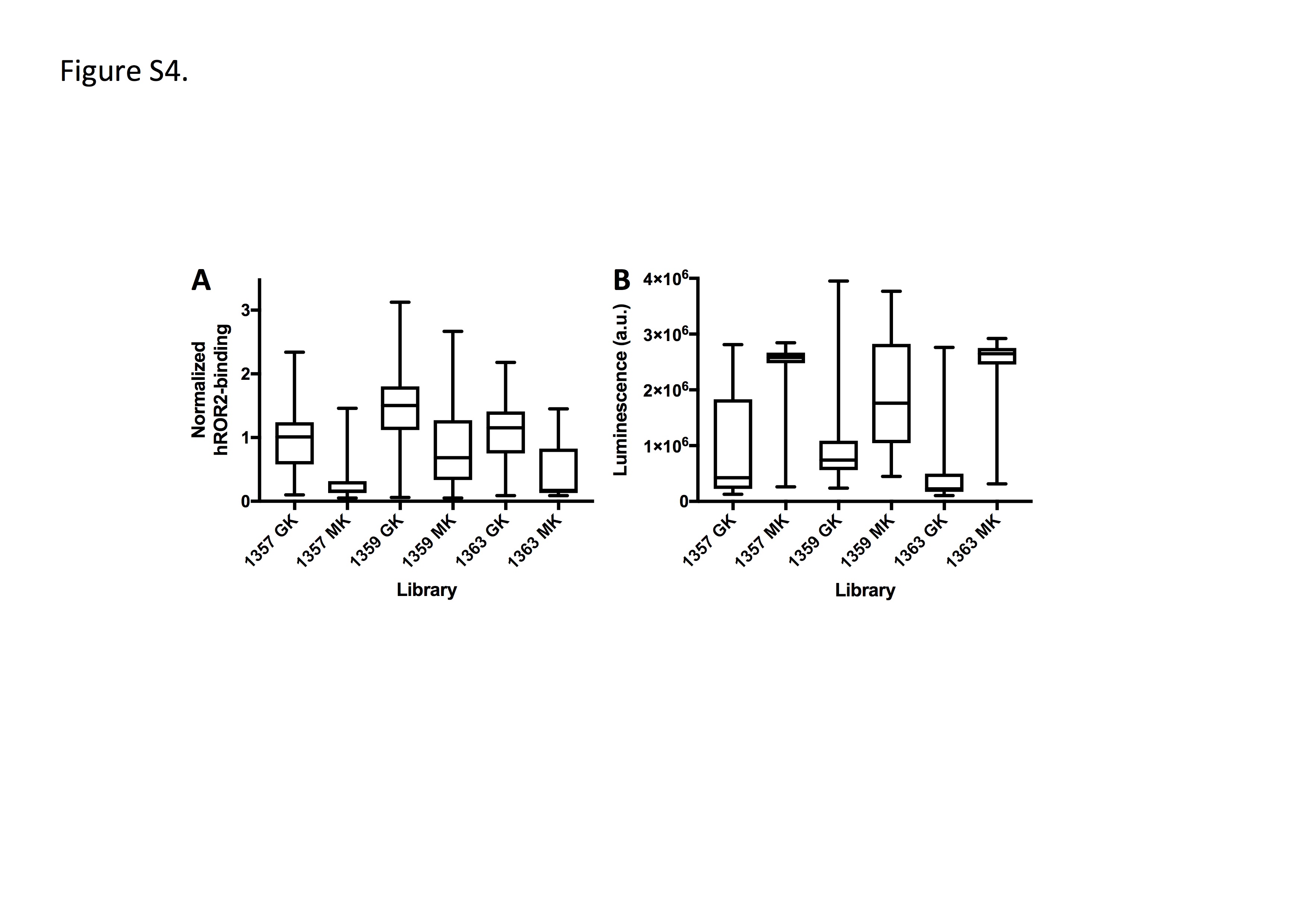

Supplement: Figure S4 — Direct screening of supernatants from sorted clones allows identification of hROR2-binding clones with high potency as an ADC. (A) Supernatants from sorted single-cell cellular clones of the (GK) and (MK) libraries (GK = VH sequences cloned from IgGs, MK = VH sequences cloned from IgMs based on different reverse primers used) derived from mice 1357, 1359, and 1363 were functionally screened for IgG levels and hROR2-ECD-Twin-Strep binding by ELISA in a single-well measurement. Normalized hROR2 binding was expressed as the ratio of OD = 490 nm hROR2-ECD-Twin-Strep binding and OD = 490 nm IgG expression for all clones from the different libraries and is shown as box-plots. (B) All supernatants were also assessed for potency as an ADC using a secondary ADC assay. For this, EMT6-hROR2 cells were incubated with clonal supernatant without normalization for IgG levels for 30 min, before addition of an anti-human Fcγ coupled via a cleavable linker to PNU-159682. Viable cells were quantified following a 3 days incubation using a luminescence-based cell viability assay. Lower luminescence values in the box-plots indicate more potent killing. [file Image_4.JPEG]

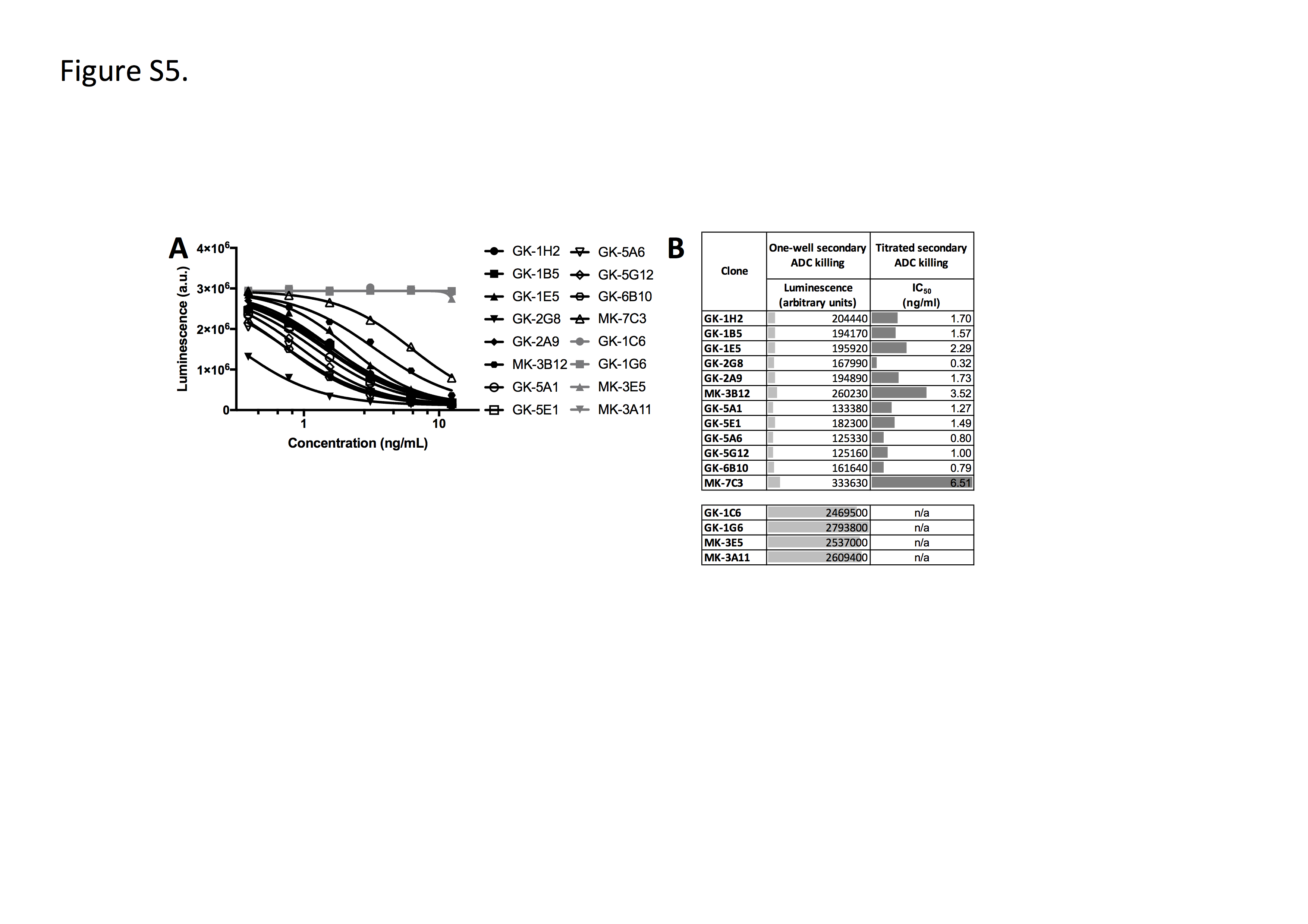

Supplement: Figure S5 — Validation of in vitro killing potency of selected clones from functional ADC screening. Twelve clonal L11 supernatants with potent in vitro killing and four supernatants with poor in vitro killing (GK-1C6, GK-1G6, MK-3E5, and MK-3A11) were selected for testing in a secondary ADC assay using a range of defined concentrations to confirm their in vitro cell killing potency. To do so, IgG levels of the supernatants were quantified by ELISA and IgG concentration in all supernatants was adjusted to the lowest expressor. EMT6-hROR2 cells were incubated with 2-fold serial dilutions of these normalized clonal supernatants for 30 min, followed by the addition of an anti-human Fcγ coupled via a cleavable linker to PNU-159682. After a 3 days incubation, viable cells were quantified using a luminescence-based cell viability assay. (A) Viability of the EMT6-hROR2 cells plotted in arbitrary units (a.u.) of luminescence on the y-axis as a function of the IgG concentration in the supernatants on the x-axis. Clonal supernatants that had potent or poor in vitro cell killing potency in the initial functional ADC screening are shown in black or gray, respectively. (B) shows luminescence values that had been determined in the one-well secondary ADC assay during functional ADC screening compared to the IC50 values which were determined in the secondary ADC assay using serial dilutions of normalized supernatants for the same clonal supernatants. IC50 values were calculated using a four-parameter curve fitting model in GraphPad Prism. n/a indicates IC50 values that could not be calculated due to a lack of in vitro killing. [file Image_5.JPEG]

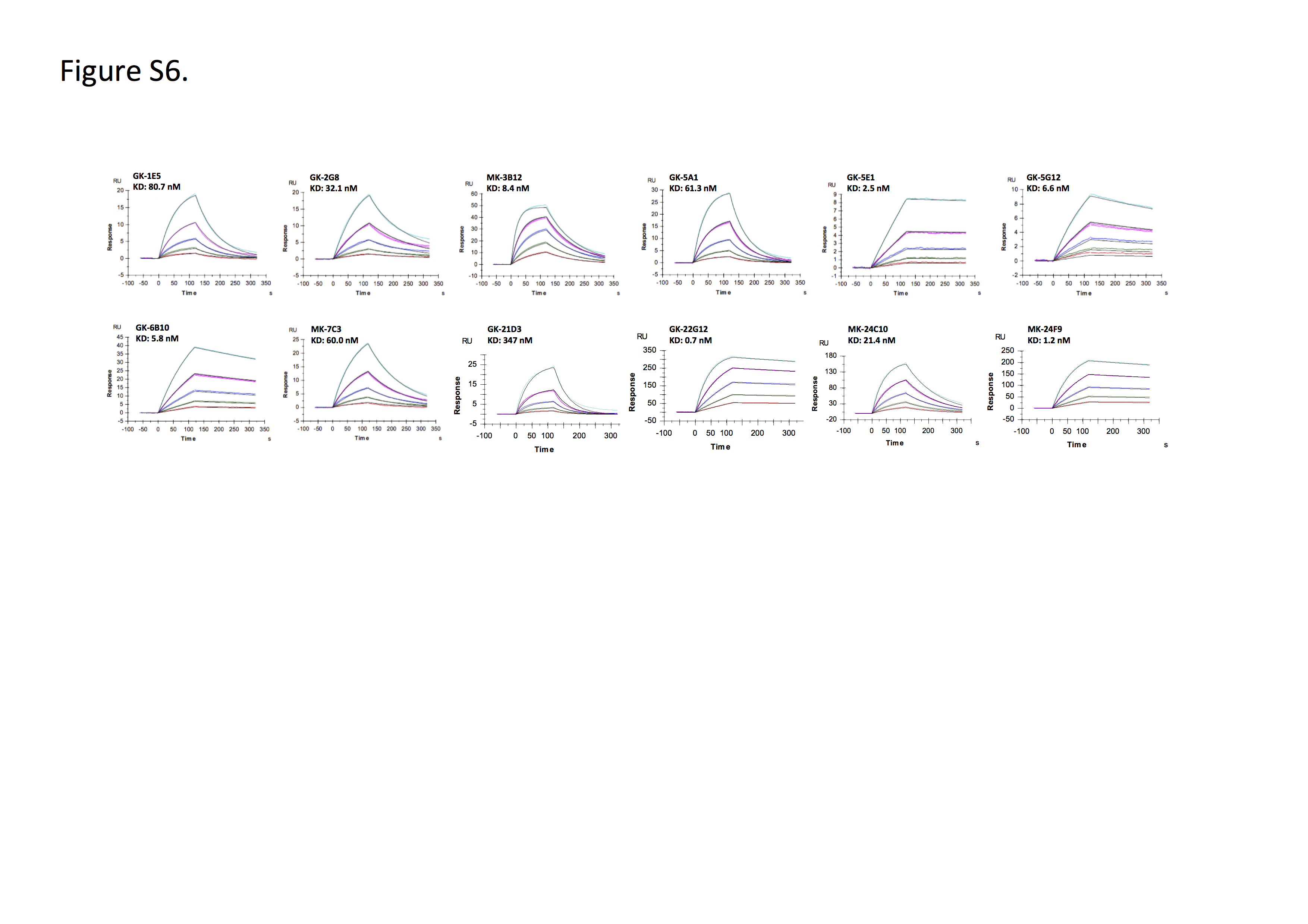

Supplement: Figure S6 — SPR sensorgrams of anti-hROR2 antibodies. Affinities were measured by multi-cycle SPR on a Biacore T200 instrument (GE Healthcare). Antibodies were captured by Protein A or G immobilized on a CM5 sensor chip, followed by the addition of hROR2-ECD-Twin-Strep used as 2-fold serial dilutions ranging from 40 to 2.5 nM. KD values as a measure of binding affinity are indicated. [file Image_6.JPEG]

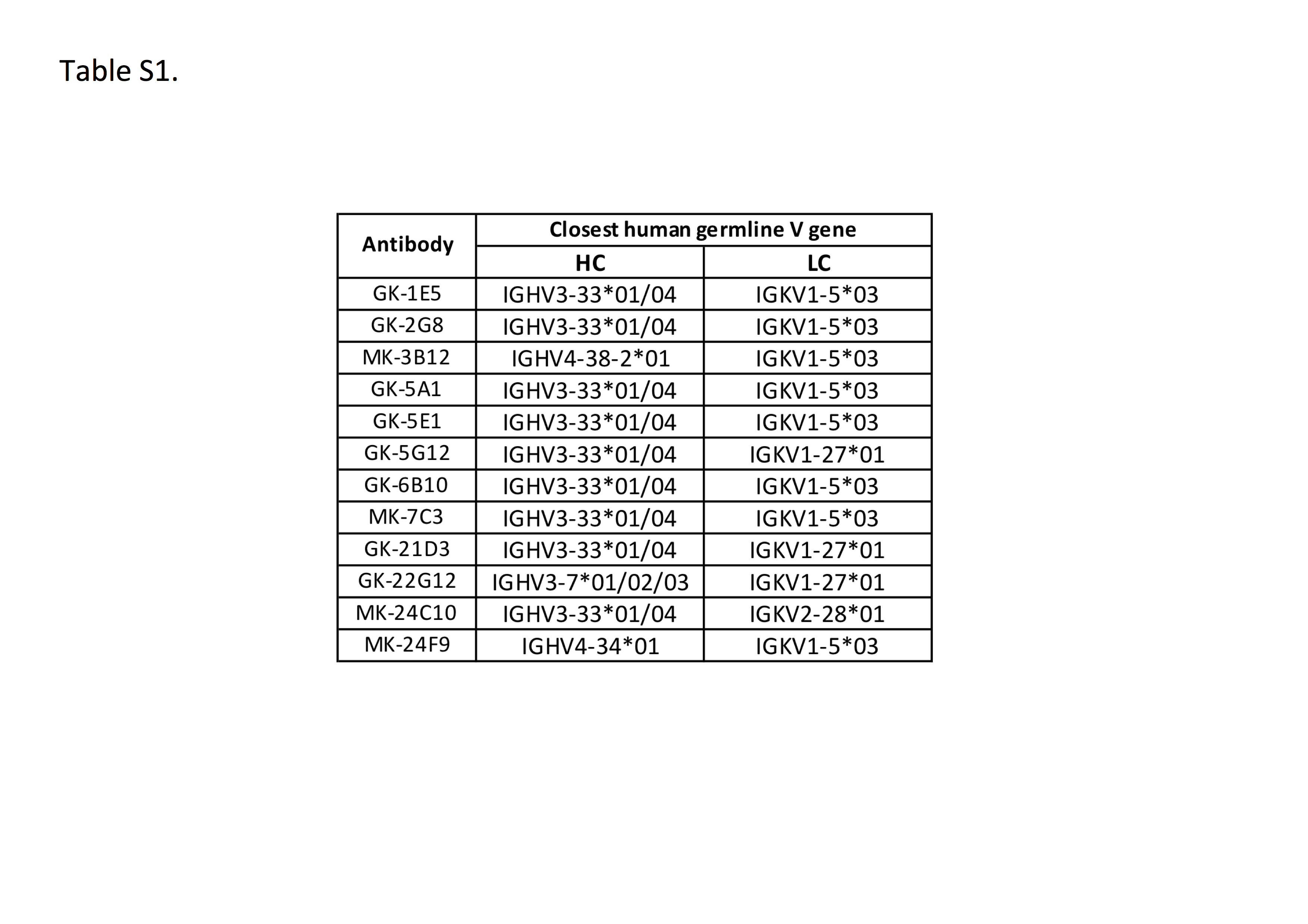

Supplement: Table S1 — Germline V gene usage of identified anti-hROR2-clonotypes. The closest human germline V gene sequences of heavy (HC) and light chain (LC) were identified in silico using IgBLAST. [file Image_7.JPEG]

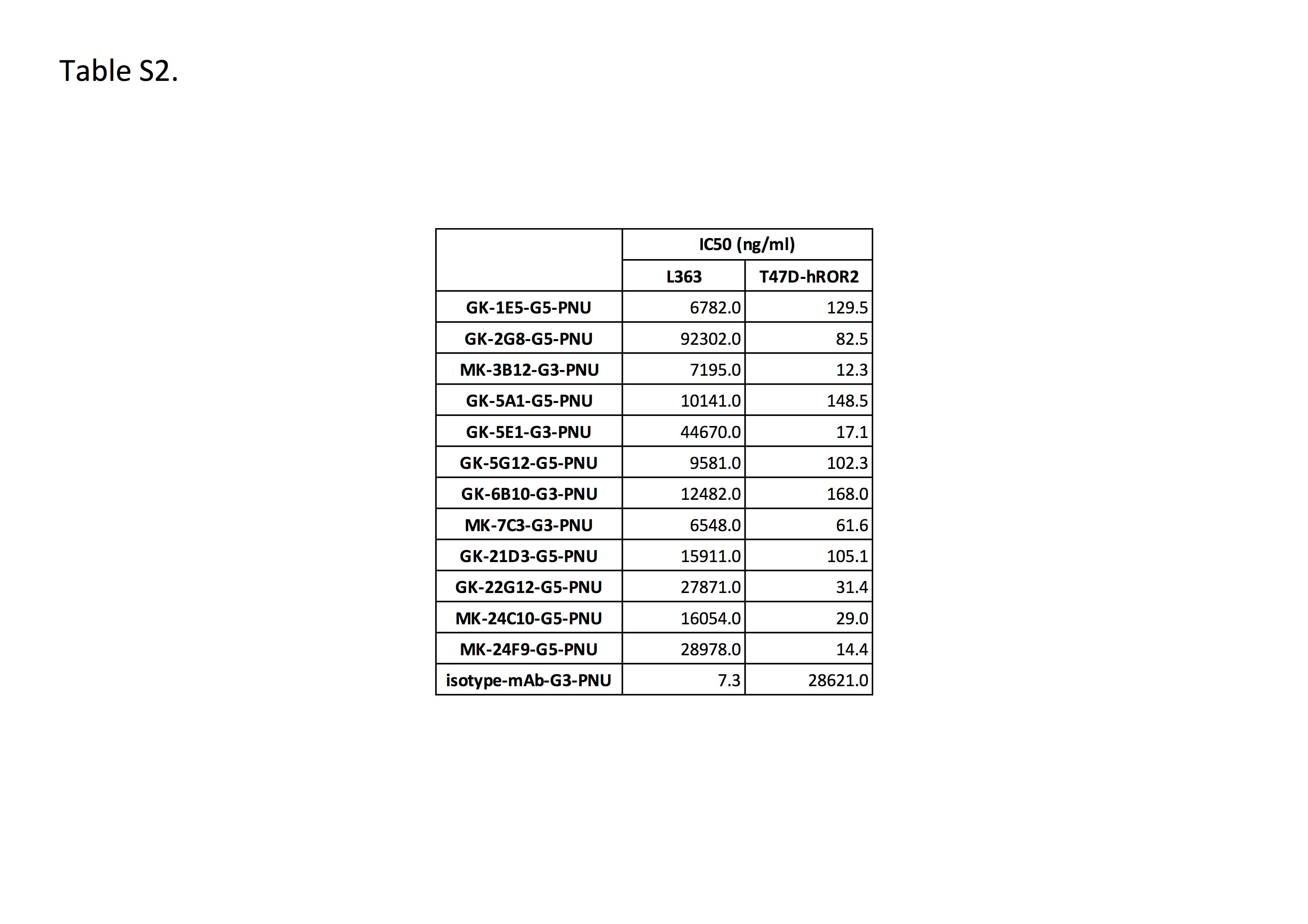

Supplement: Table S2 — In vitro cell killing by anti-hROR2-ADCs. IC50 values (ng/ml) reported here represent the IC50 values of the hROR2-specific ADCs tested for their cell killing activity on hROR2-negative L363 and hROR2-high EMT6-hROR2 cell lines in Figure 7. IC50 values were calculated from the mean of two replicates using a four-parameter curve fitting model in GraphPad Prism. [file Image_8.JPEG]
